# The maturase HydF enables [FeFe] hydrogenase assembly via transient, cofactor-dependent interactions

**DOI:** 10.1074/jbc.RA119.011419

**Published:** 2020-07-03

**Authors:** Brigitta Németh, Henrik Land, Ann Magnuson, Anders Hofer, Gustav Berggren

**Affiliations:** 1 Department of Chemistry–Ångström Laboratory, Uppsala University, Uppsala, Sweden; 2 Department of Medical Biochemistry and Biophysics, Umeå University, Umeå, Sweden

**Keywords:** metalloenzyme, metal ion–protein interaction, mass spectrometry (MS), Fourier transform IR (FTIR), hydrogenase, cofactor, chaperone, scaffold, iron-sulfur protein, protein–protein interaction, metallo-cofactor assembly

## Abstract

[FeFe] hydrogenases have attracted extensive attention in the field of renewable energy research because of their remarkable efficiency for H_2_ gas production. H_2_ formation is catalyzed by a biologically unique hexanuclear iron cofactor denoted the H-cluster. The assembly of this cofactor requires a dedicated maturation machinery including HydF, a multidomain [4Fe4S] cluster protein with GTPase activity. HydF is responsible for harboring and delivering a precatalyst to the apo-hydrogenase, but the details of this process are not well understood. Here, we utilize gas-phase electrophoretic macromolecule analysis to show that a HydF dimer forms a transient interaction complex with the hydrogenase and that the formation of this complex depends on the cofactor content on HydF. Moreover, Fourier transform infrared, electron paramagnetic resonance, and UV-visible spectroscopy studies of mutants of HydF show that the isolated iron-sulfur cluster domain retains the capacity for binding the precatalyst in a reversible fashion and is capable of activating apo-hydrogenase in *in vitro* assays. These results demonstrate the central role of the iron-sulfur cluster domain of HydF in the final stages of H-cluster assembly, *i.e.* in binding and delivering the precatalyst.

Hydrogenases are metalloproteins that catalyze the reversible interconversion of protons and electrons to molecular hydrogen and are often divided into different classes based on the nature of the catalytic cofactor (1, 2). In the case of [FeFe] hydrogenases (HydA), the reaction is catalyzed by a hexanuclear iron complex, the H-cluster. This cofactor is composed of a canonical four-cysteine coordinated FeS cluster ([4Fe4S]_H_) coupled to a low-valence (Fe_2_^I,I/II,II^) diiron site, the [2Fe] subsite. The latter complex is coordinated by carbonyl and cyanide ligands, as well as a bridging dithiolate ligand (adt = ^−^SCH_2_NHCH_2_S^−^). The biosynthesis of the H-cluster is a complex, multistep process, during which the [4Fe-4S]_H_-cluster is first assembled by the standard house-keeping FeS cluster machinery (3–5). The active hydrogenase is then generated through the combined activities of at least three [FeFe] hydrogenase-specific maturation enzymes, denoted HydE, HydG, and HydF, responsible for the synthesis and insertion of the [2Fe] subsite (Fig. 1) (6). More specifically, HydE and HydG are radical SAM enzymes responsible for the construction of a [2Fe] subsite precatalyst on HydF, a scaffold protein that delivers the cofactor to the hydrogenase (7–17). A combination of FTIR spectroscopy, biochemical assays, biomimetic model chemistry, and DFT calculations has revealed that this precatalyst is strikingly similar to the synthetic [2Fe] subsite mimic [Fe_2_(adt)(CO)_4_(CN)_2_]^2−^ ([2Fe]^adt^) (14, 18–20).

**Figure 1.**
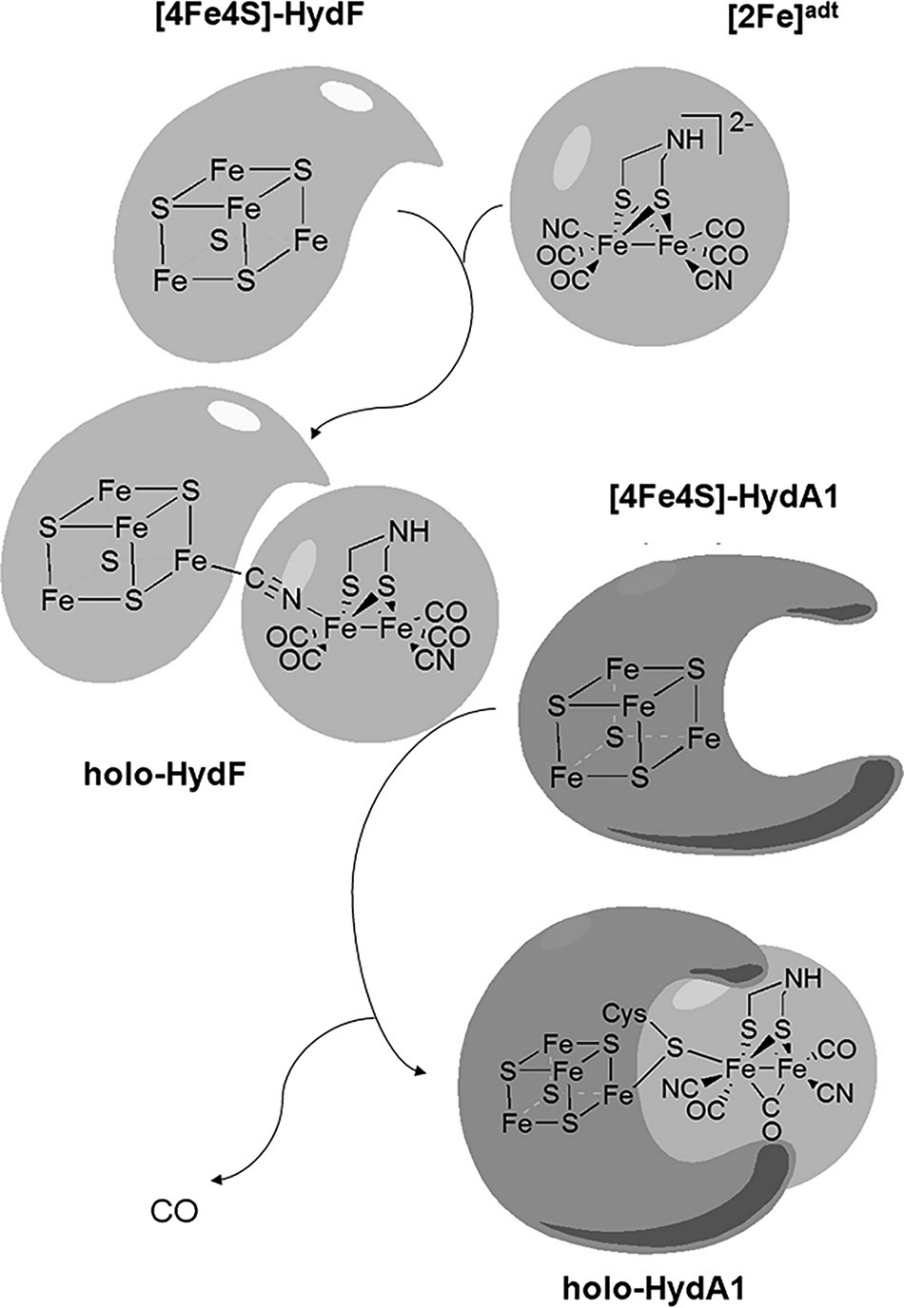
Schematic representation of the metal cofactors in HydF and HydA and the maturation of [FeFe] hydrogenase by HydF.

The HydF protein is a GTPase containing a [4Fe-4S] cluster (21). More specifically, X-ray crystallography studies have shown that HydF has three distinct domains, a GTPase domain, a dimerization domain, and a FeS cluster binding domain (18, 22). Point mutations in the walker-loop of the GTPase domain, as well of the FeS binding cysteine residues, have been shown to impair HydF function and prevent assembly of a functional [FeFe] hydrogenase under *in vivo* conditions (6). The GTPase function appears to be critical during the interaction between HydF and HydE as well as HydG, *i.e.* upstream in the [2Fe] precatalyst biosynthetic pathway (23). The GTP hydrolysis plays a key role in the dissociation of HydF from HydE and HydG, but it does not seem to influence the interaction with HydA (17). The dimerization domain exhibits a hydrophobic surface that leads to a strong hydrophobic interaction between two copies of the HydF polypeptide, both for the apo-form as well as for the FeS cluster-containing form ([4Fe-4S]-HydF) (18, 22). The third domain contains three conserved cysteines and a glutamate residue responsible for the coordination of the [4Fe-4S] cluster on HydF. The function of the [4Fe-4S] cluster is still not fully elucidated, but spectroscopic studies of HydF from *Thermotoga maritima* suggest that it is involved in binding of the precatalyst to HydF. This is further supported by recent single-point-mutation studies of HydF from *Thermosipho melanesiensis* (14, 18). Still, crystallographic support for this model is lacking, and the importance of the different domains for binding the [2Fe] precatalyst and for the HydF–HydA interaction remains unknown. The dependence on efficient coexpression of HydG and HydE for generation of the active form of HydF, *i.e.* a form carrying the precatalyst, has complicated studies of this protein. However, it was recently shown how this can be circumvented: HydF expressed in the absence of HydE and HydG can be loaded with synthetic analogues of the [2Fe] precatalyst to generate semisynthetic forms of the protein indistinguishable from HydF expressed in the presence of HydE and HydG (14, 18, 19). Whereas these biophysical and crystallographic studies on both “native” and semisynthetic forms have provided an increasingly detailed view of the HydF protein, the mechanism by which it interacts with, and activates, the hydrogenase enzyme is still, to a large extent, unknown.

Here, we report on the quaternary structure of the HydF protein and the HydF–HydA interaction complex as a function of metal content in the two proteins, elucidated using primarily gas-phase electrophoretic macromolecule analysis (GEMMA), a technique highly suitable for probing weak protein interaction complexes (24, 25). The GEMMA reveals a transient interaction between the two proteins. Moreover, the influence of the different domains of HydF is further probed through the preparation of truncated forms of HydF, lacking either the dimerization domain (HydFΔD) or both the dimerization as well the GTPase domain (HydFΔDG) (see the supporting information for amino acid sequences). Both truncated constructs retained the capacity to bind the [2Fe]^adt^ precatalyst, verifying the role of the FeS cluster domain for harboring the diiron complex prior to transfer to apo-HydA. Moreover, neither the GTPase nor the dimerization domain was found to be critical for releasing the precatalyst once bound to HydF, as both HydFΔD and HydFΔDG could activate apo-hydrogenase under *in vitro* conditions.

## Results

### Quaternary structure of HydA1, HydF, and their interaction complex

To monitor the effect of cofactor loading on the quaternary structure of HydF and HydA, as well as their interaction behavior, a series of samples of the two proteins was prepared with varying metal cofactor content. As the maturation proteins are well conserved, we utilized HydF from *T. maritima* and the HydA1 [FeFe] hydrogenase from *Chlamydomonas reinhardtii* as our model proteins (6, 26, 27). These two proteins are well characterized *in vitro*, and it has been previously established that *T. maritima* HydF is capable of activating HydA1 (1, 14, 28). More specifically, both HydF and HydA1 were expressed and purified under aerobic conditions, and metal-free apo-proteins (apo-HydF and apo-HydA1) were prepared via chelation of residual metal ions under reducing anaerobic conditions. The FeS clusters were then reconstituted by following literature protocols, with minor modifications, to generate [4Fe-4S]-HydF and [4Fe4S]-HydA1 (29, 30). Finally, semisynthetic forms of holo-HydF and holo-HydA1 were prepared by treating [4Fe-4S]-HydF and [4Fe-4S]-HydA1 with a 12-fold excess of [2Fe]^adt^, as previously described (14, 18, 31).

### Examining quaternary structure of the apo-proteins by size-exclusion chromatography and GEMMA

**Figure 2.**
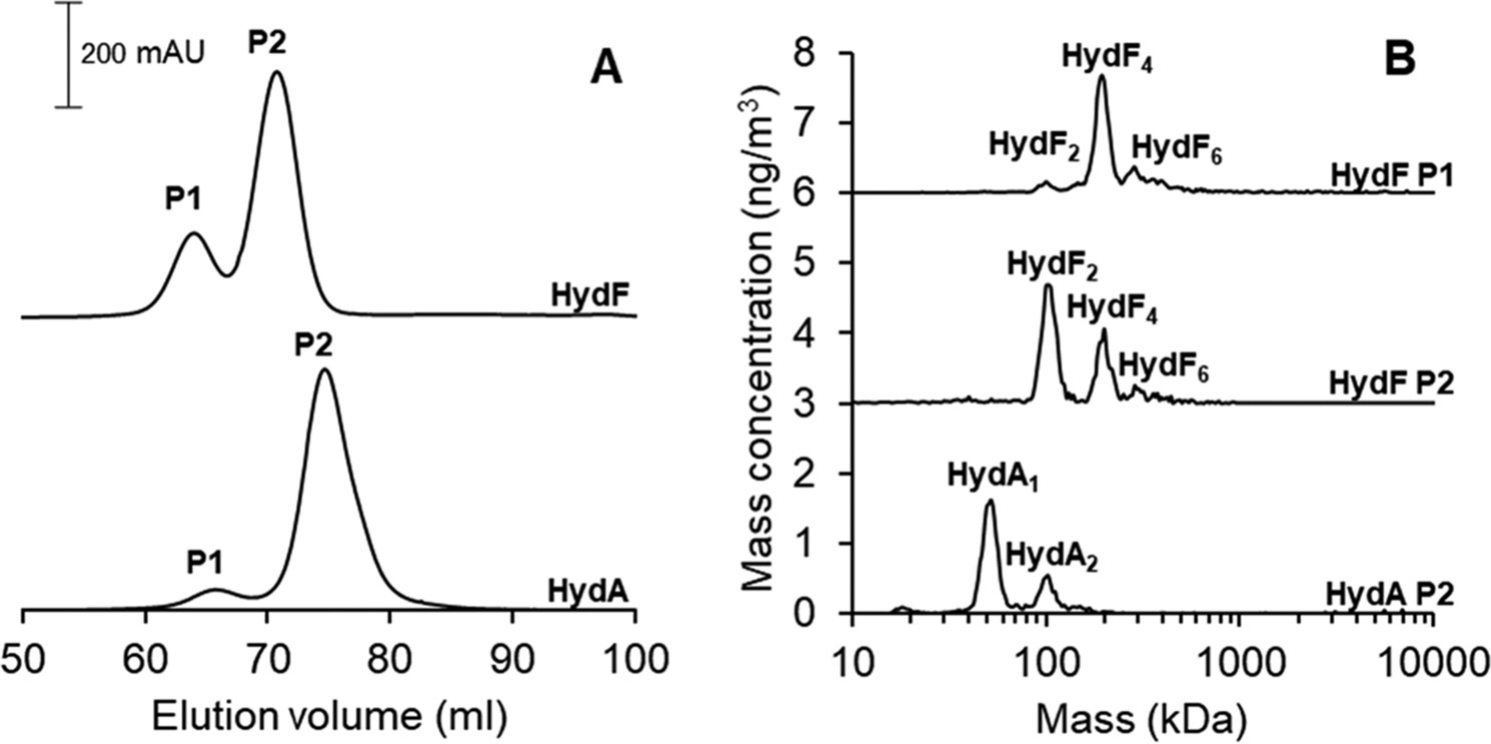
*A*, SEC chromatograms of the apo-HydF (*top*) and apo-HydA1 (*bottom*). Running conditions were 0.8 ml/min flowrate, 50 mm Tris-HCl, pH 8.0, 150 mm NaCl. *B*, the corresponding GEMMA spectra recorded for the different fractions of the apo-HydF and apo-HydA1 proteins obtained from SEC. Running conditions were 0.02 µg/µl protein in 20 mm ammonium acetate and 0.005% Tween 20.

The size-exclusion chromatography was performed on the apo-proteins on a Superdex-200 column, directly following the demetallization procedure. Samples of apo-HydF featured a peak at 63 ml (P1) together with a larger peak at 70 ml (P2) (Fig. 2*A*, *top*). In the case of apo-HydA1, a similar, albeit slightly shifted, chromatogram was observed, displaying a dominant peak (P2) at 75 ml, preceded by a smaller peak at 65 ml (P1) (Fig. 2*A*, *bottom*).

The corresponding elution fractions were collected and concentrated separately. After buffer exchange, the proteins were measured with wide-scan GEMMA (1 kDa to 10 MDa). The GEMMA spectrum of the P2 fraction of apo-HydA1 exhibits a peak close to 50 kDa (Fig. 2*B*, spectrum HydA P2), together with an additional minor peak at 100 kDa. The 50-kDa peak corresponds well to a monomeric form of HydA1 (theoretical mass, 49 kDa), in agreement with earlier studies showing that the enzyme is monomeric in solution. In the case of apo-HydF, the P1 fraction exhibits a large peak at ∼200 kDa, together with two other minor peaks at 100 and 300 kDa (Fig. 2*B*, spectrum HydF P1). The molecular weight of the HydF protein is ≈45 kDa; thus, we assign the peaks to tetrameric, dimeric, and hexameric forms of the HydF protein. We conclude that the first peak (P1) of the size-exclusion chromatogram represents the tetrameric form of apo-HydF.

The GEMMA spectrum of the P2 fraction of apo-HydF is more complex. The strongest peak in the spectrum appears around 100 kDa, in agreement with the successful isolation of dimeric apo-HydF (Fig. 2*B*, spectrum HydF P2). However, significant peaks are also observable at 200 and 300 kDa, suggesting that the dimer form of apo-HydF is unstable on the time scale of the experiment. This conclusion is further supported by time-dependent GEMMA studies. The higher-molecular-weight peaks increased in intensity in the P2 protein samples on a minute time scale, even for apo-HydF samples kept on ice under strict anaerobic conditions (data not shown).

### The effect of cofactor content on the quaternary structure of isolated HydF and HydA1

The crystal structures of both apo-HydF and [4Fe-4S]-HydF clearly support the dimeric nature of the protein. In addition, both reported structures reveal the formation of a tetrameric, or dimer-of-dimers, structure. In the apo-protein, the dimer-of-dimers is formed via disulfide bridges involving the cysteines in the FeS cluster domain (22). These residues are involved in metal ligation in [4Fe-4S]-HydF, preventing their formation of disulfide bridges. In the latter case, the formation of the dimer-of-dimers is instead potentially a result of crystal packing (18). To clarify the influence of the metal cofactors on the quaternary structure of HydF, holo-HydF was assembled in a stepwise fashion, and apo-HydF, [4Fe-4S]-HydF, and holo-HydF each were analyzed by GEMMA. Likewise, apo-HydA1, [4Fe-4S]-HydA1, and holo-HydA1 were prepared and analyzed in parallel assays.

**Figure 3.**
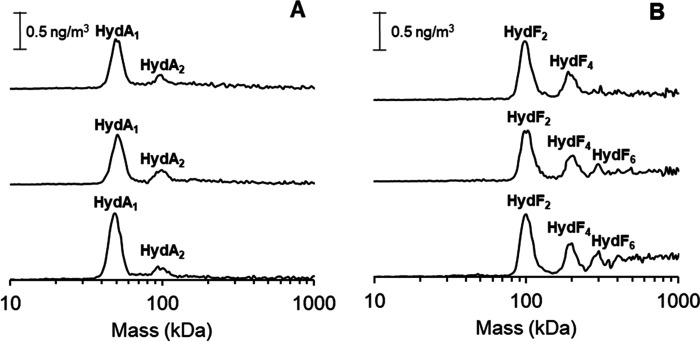
**GEMMA spectra recorded on HydF and HydA1 proteins with different cofactor loadings.** *A*, HydA1 (*top*, holo-HydA1; *middle*, [4Fe4S]-HydA1; *bottom*, apo-HydA1); *B*, HydF (*top*, holo-HydF; *middle*, [4Fe4S]-HydF; *bottom*, apo-HydF). The protein concentration in all samples were 0.02 µg/µl in 20 mm ammonium acetate and 0.005% Tween-20.

As seen in Fig. 3*A*, HydA1 remained predominantly monomeric regardless of cofactor loading. In the case of HydF, the dimeric form of the protein dominated the mass spectrum, with a substantial fraction of tetrameric forms also discernable in all cases. However, a significant reduction in the higher-molecular-mass species (hexamers and larger) was observed for the metal-loaded forms. These results show that there is no drastic change in the appearance of dimeric and tetrameric forms of HydF as a function of cofactor content. Apart from the FeS cluster binding cysteines, there is one additional cysteine residue present in the protein. However, the latter cysteine is relatively buried in the GTPase domain and unlikely to be involved in interprotein disulfide formation (see Fig. S2 and S3 for predicted *Thermotoga maritima* HydF [TmHydF] structures). Thus, the observation that a fraction of metal loaded HydF also exists in tetrameric form shows that disulfide formation is not the sole contributor to the formation of the dimer-of-dimer HydF complex. Instead, the tetrameric form of HydF is likely to be attributable to interactions between the dimerization and FeS cluster binding domains of two HydF dimers, as suggested from the XRD data of [4Fe4S]-HydF (18). This complex could, in turn, promote the formation of interdimer disulfide bridges, providing a strong covalent linkage (22). Still, the formation of multimeric forms can be reduced by introducing the [4Fe4S] and [2Fe]^adt^ cofactors, supporting the notion that they, to a large extent, arise from disulfide formation via the cluster binding cysteine residues.

### The effect of cofactor content on the HydF–HydA1 interaction

The final stages of H-cluster assembly are initiated with the transfer of the precatalyst from HydF to HydA1. To probe the structure and stability of the expected interaction complex between the two proteins, mixtures of HydF and HydA1 were prepared and analyzed by GEMMA. More specifically, apo-HydA1, [4Fe-4S]-HydA1, or holo-HydA1 was mixed with apo-HydF, [4Fe-4S]-HydF, or holo-HydF, and the masses of the resulting interaction complexes were recorded.

**Figure 4.**
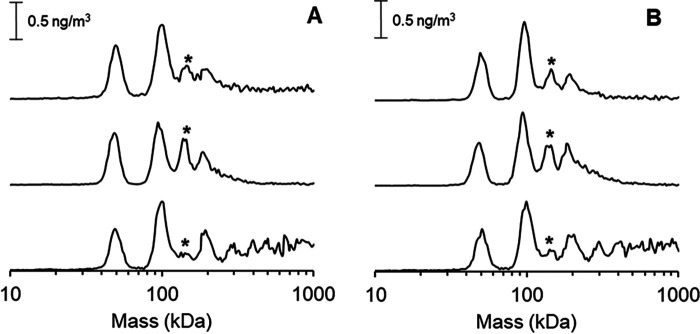
**GEMMA spectra recorded on combinations of HydF and HydA1 proteins with different cofactor content.** *A*, apo-HydA1 interaction with different cofactor containing forms of HydF (*top*, holo-HydF; *middle*, [4Fe4S]-HydF; *bottom*, apo-HydF). *B*, [4Fe4S]-HydA1 interaction with different cofactor containing forms of HydF (*top*, holo-HydF; *middle*, [4Fe4S]-HydF; *bottom*, apo-HydF). The 150-kDa peak representing the HydF_2_–HydA1 interaction complex is indicated with an asterisk. The experiments were performed with 0.02 µg/µl each protein (total protein concentration, 0.04 µg/µl) in 20 mm ammonium acetate and 0.005% Tween-20.

A distinct peak was observed at ≈150 kDa when HydA1 was mixed with either [4Fe-4S]-HydF or holo-HydF (Fig. 4, *middle* and *top spectra*, and Fig. S4). This peak most likely is attributable to an interaction complex consisting of a HydF dimer and a monomeric HydA1 protein (HydF_2_HydA1). In addition, peaks attributable to monomeric HydA1, as well as dimeric and tetrameric HydF, were clearly visible in all spectra. Similar trends were observed when either apo-HydA1 (Fig. 4*A*) or [4Fe-4S]-HydA1 (Fig. 4*B*) were present. Interestingly, the interaction peak at 150 kDa changed in response to the cofactor content of HydF. In the presence of metal-loaded HydF, *i.e.* either [4Fe4S]-HydF or Holo-HydF, the peak at 150 kDa was clearly observable, whereas the larger oligomeric forms of HydF could not be discerned (Fig. 4, *top* and *middle spectra*). Mixtures of apo-HydF and HydA1, on the other hand, resulted in complicated spectra (Fig. 4, *bottom*, and Fig. S4), and only traces of a feature attributable to a HydF_2_–HydA1 interaction could be discerned. Instead, the latter spectra mainly displayed peaks arising from monomeric HydA1 and dimeric HydF. Moreover, additional peaks attributable to the tetrameric form of HydF, as well as larger HydF protein complexes, were clearly visible in the spectra, in agreement with studies of the apo-HydF protein in isolation (Fig. 3*B*). It should be noted that partial formation of holo-HydA1 can occur in assays with holo-HydF. However, on the minute time scale of these assays, this should only influence a minor fraction of the [4Fe4S]-HydA1 sample (19). Still, this latter effect could explain the apparent decrease in HydA affinity of holo-HydF compared with that of [4Fe4S]-HydF (Fig. S5).

**Figure 5.**
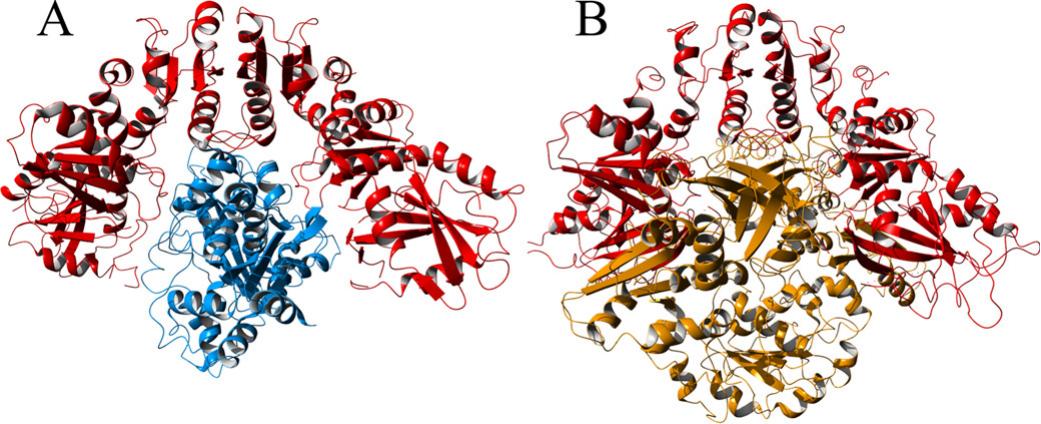
*A*, a rigid-body protein-protein docking model of the complex between dimeric [4Fe4S]-HydF (*red*) and [4Fe4S]-HydA1 (*blue*). Models are based on reported crystal structures (PDB entries 5KH0 and 3LX4). *B*, crystal structure of the tetrameric form of apo-HydF (one HydF dimer shown in *red*, analogously to *panel A*, and one in *brown*), revealing a blockage of the interaction crevice (PDB entry 3QQ5).

In summary, the GEMMA data support a model where a dimer of HydF interacts with HydA1, and that this binding is at least partially determined by the cofactor loading of HydF. The reverse scenario, with a monomeric HydF interacting with a dimeric HydA, is possible but unlikely, in light of the observations reported here and earlier, that HydF always exists in dimeric or larger forms in solution and *in cristallo* (18, 21, 22, 32). This dimerization behavior is attributable to the very strong hydrophobic interaction between the dimerization domains, which exhibits a very hydrophobic surface (Fig. S3). No distinct interaction peak was observed that suggested a complex between a tetramer of HydF and HydA1. However, it should be noted that even for the metal-loaded forms of HydF, the 150-kDa interaction peak is relatively small, and predominantly isolated HydF and HydA1 remain in solution, suggesting that the HydF_2_–HydA1 interaction is transient and preventing a quantification of the binding constants. The observation that the cofactor content of HydF has a stronger effect on the interaction than the cofactor content of HydA1 can be rationalized on a structural basis using the reported crystal structures. In HydF, the [4Fe-4S]-cluster is surface exposed toward the interior cavity of the dimer. Thus, its presence is likely to influence the interaction surface, assuming that the FeS cluster-binding domain comes in close proximity to the HydA1 protein. Conversely, the [4Fe-4S]-cluster is more buried in the case of HydA1; thus, it is unlikely to significantly alter the surface properties of the HydA1 protein (3, 18, 22).

### In silico protein–protein docking

To further investigate the interaction between HydF and HydA1, a rigid-body protein–protein docking analysis was performed using ClusPro 2.0 (33–35). The docking was made using crystal structures of [4Fe-4S]-HydA1 (PDB entry 3LX4)(3) and dimeric [4Fe-4S]-HydF from *T. melanesiensis* (PDB entry 5KH0) (18). As previously observed, the HydF dimer forms a cavity where the [4Fe-4S] clusters are exposed (18). The protein–protein docking analysis revealed that HydA1 seemingly fits nicely into this cavity (Fig. 5 and Fig. S6; see also reference 37). To investigate the possibility for HydA1 to interact with the tetrameric form of HydF, a comparison was made between the docked HydA1-HydF complex and the published crystal structure of tetrameric *T. neapolitana* HydF (PDB entry 3QQ5) (22). The results show that the two dimers of HydF forming the tetramer are interacting via each dimer's respective cavity, leaving no room for HydA1 (Fig. 5*B*). This strengthens our finding that HydA1 only interacts with the HydF dimer and not the tetramer, as the binding site in the tetramer is effectively closed off by the second HydF dimer.

**Figure 6.**
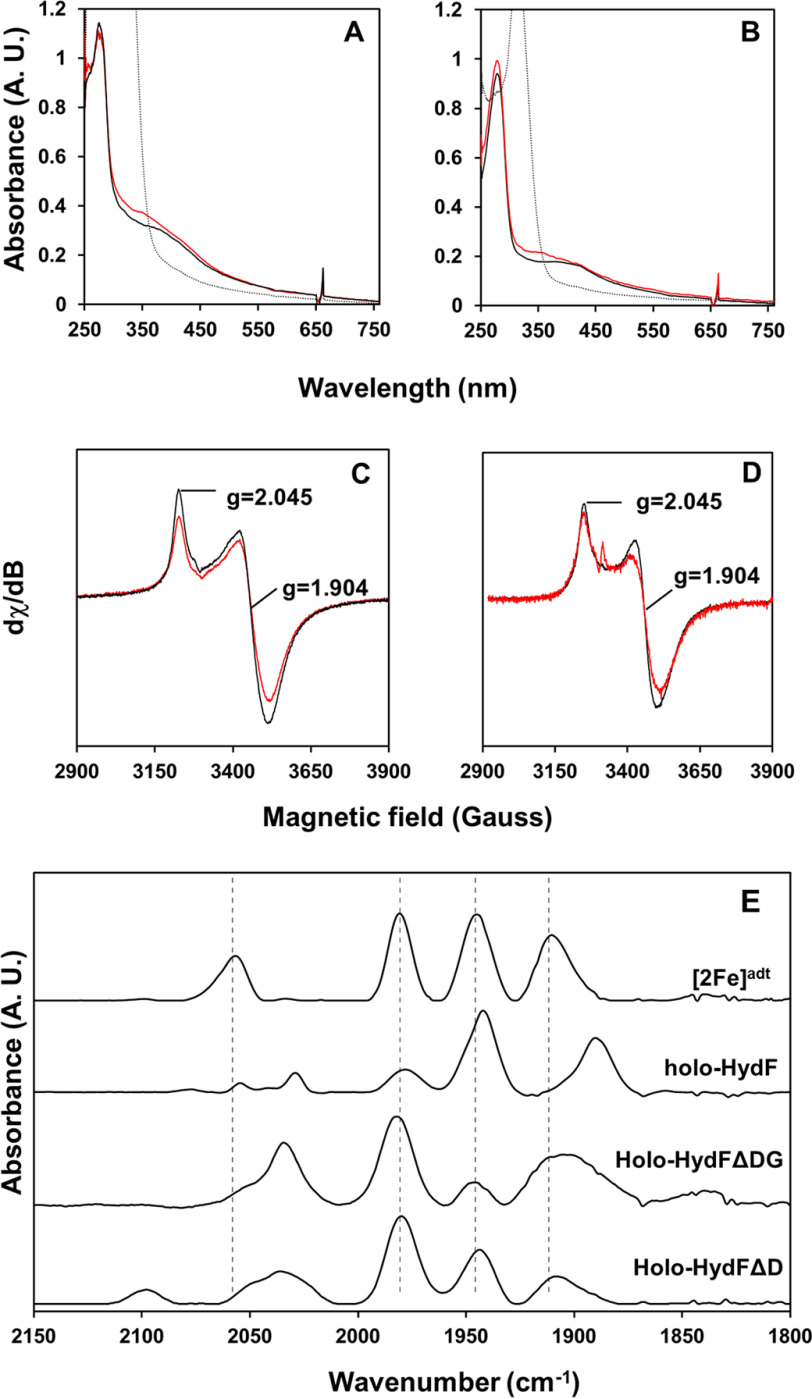
**Spectroscopic characterization of the truncated HydF proteins.** *A* and *B*, UV-visible spectra of HydFΔD (*A*) and HydFΔDG (*B*). Shown are the reconstituted ([4Fe-4S]^2+^) form of the HydF variants (*black spectra*), the [2Fe]^adt^ cofactor-loaded holo-forms ([2Fe]^adt^-[4Fe-4S]^2+^) (*red spectra*), and the Na-DT reduced ([4Fe-4S]^+^) forms (*dashed spectra*). The samples were prepared in a buffer containing 100 mm Tris-HCl and 300 mm KCl with a protein concentration of 100 μm (HydFΔD) or 50 μm (HydFΔDG). Na-DT (0.5 mm) was added to generate the reduced samples. *C* and *D*, low-temperature EPR spectra of the reduced forms of HydFΔD (200 μm) (*C*) and HydFΔDG (200 μm) (*D*). Shown are both reconstituted (*black spectra*) and [2Fe]^adt^-loaded forms (*red spectra*), and observed *g*-values are indicated. *E*, FTIR spectra of the [2Fe]^adt^-loaded proteins, holo-HydFΔD and HydFΔDG. Spectra recorded for [2Fe]^adt^ and holo-HydF are displayed for comparison, and the peak positions of [2Fe]^adt^ are indicated with vertical dashed lines. The EPR spectra were recorded at 10 K, 1 mW microwave power, 10-Gauss modulation amplitude, and 100-kHz modulation frequency. The microwave frequency was 9.28 GHz. The FTIR spectra were recorded at room temperature, and the samples were prepared in a solution containing approximately 2.5 mm protein, 100 mm Tris-HCl, pH 8.0, 300 mm KCl.

### Preparation and reactivity of truncated HydF

The aforementioned GEMMA study underscores the transient nature of the HydF–HydA1 interaction. To further elucidate the importance of the different domains specifically for the final step in the maturation processes, we prepared truncated forms of HydF and compared their capacity to not only bind the [2Fe]^adt^ precatalyst but also to transfer it to [4Fe-4S]-HydA1. Although crystallographic verification is lacking, biochemical and spectroscopic characterizations of holo-HydF support a model in which the precatalyst is bound to the FeS-cluster domain (14, 18, 20). Consequently, we retained this domain and prepared two truncated forms, in which either the dimerization domain (HydFΔD) or both the dimerization domain and the GTPase domain had been removed (HydFΔDG). The HydFΔD construct was readily isolated by affinity chromatography following recombinant expression in *Escherichia coli*. Likewise, the HydFΔDG protein was isolated in good yields after the addition of an N-terminal solubility tag (maltose-binding protein, MBP).

As for the full-length HydF protein, the two truncated forms were initially prepared in their apo-form and their respective [4Fe-4S] cluster reconstituted *in vitro*. The successful assembly of a [4Fe-4S] cluster in both HydFΔD ([4Fe-4S]-HydFΔD) and HydFΔDG ([4Fe-4S]-HydFΔDG) was apparent from iron quantification assays, which showed a final iron content of 3.67 ± 0.6 ([4Fe-4S]-HydFΔD) and 3.38 ± 0.25 ([4Fe-4S]-HydFΔDG), and was verified by UV-visible and EPR spectroscopy (Fig. 6). For both truncated proteins, the UV-visible spectrum showed the expected broad absorbance band around 400 nm, in agreement with an oxidized (^2+^) cluster after reconstitution (Fig. 6, *A* and *B*, *black spectra*). This band decreased in intensity upon reduction, with the concomitant appearance of an axial EPR signal attributable to the reduced [4Fe-4S]^+^ cluster (Fig. 6, *C* and *D*, *black spectra*). Thus, in both truncated proteins, the FeS cluster domain retained its capacity to form a redox-active [4Fe-4S] cluster analogous to that observed in the full-length protein.

Treating [4Fe-4S]-HydFΔD and [4Fe-4S]-HydFΔDG with an excess of [2Fe]^adt^ (12 molar equivalents), followed by purification on a desalting column, resulted in the binding of the precatalyst to the protein in both cases (the resulting proteins are denoted holo-HydFΔD and holo-HydFΔDG, respectively). As observed for the full-length holo-HydF protein (14, 18, 19), the incorporation of [2Fe]^adt^ gave rise to a new feature in the UV-visible spectrum, around 350 nm, observed for both truncated proteins (Fig. 5, *A* and *B*, *red spectra*). In further agreement with the full-length protein, the EPR spectra displayed only limited changes (Fig. 6, *C* and *D*, *red spectra*). The reduced truncated holo-proteins revealed axial signals highly similar to those of their respective [4Fe-4S]^+^ precursors.

The binding of the [2Fe]^adt^ complex was also readily observable by FTIR spectroscopy in the metal carbonyl/cyanide region (2,200–1,750 cm^−1^) of their respective FTIR spectrum (Fig. 6*E*). Overall, the FTIR spectra of the two truncated proteins were similar, and the positions of the CO bands overlap closely the bands observed for the isolated [2Fe]^adt^ complex in solution. Conversely, the cyanide band observed at ≈2,060 cm^−1^ for [2Fe]^adt^ is clearly shifted toward lower wavenumbers upon binding to HydFΔDG or HydFΔD and overlap closer with the cyanide bands of holo-HydF. In summary, the absence of the dimerization domain appears to have changed the binding pocket relative to the native protein, arguably resulting in a more solvent-exposed site. In addition, the absence of any distinct differences between the HydFΔDG and HydFΔD constructs shows that the GTPase domain is not directly influencing the environment of the cofactor. The fact that the cyanide band is still shifted in these truncated forms of HydF is in good agreement with the proposed binding of the precatalyst to the FeS cluster of HydF via a bridging cyanide ligand (14, 20).

**Figure 7.**
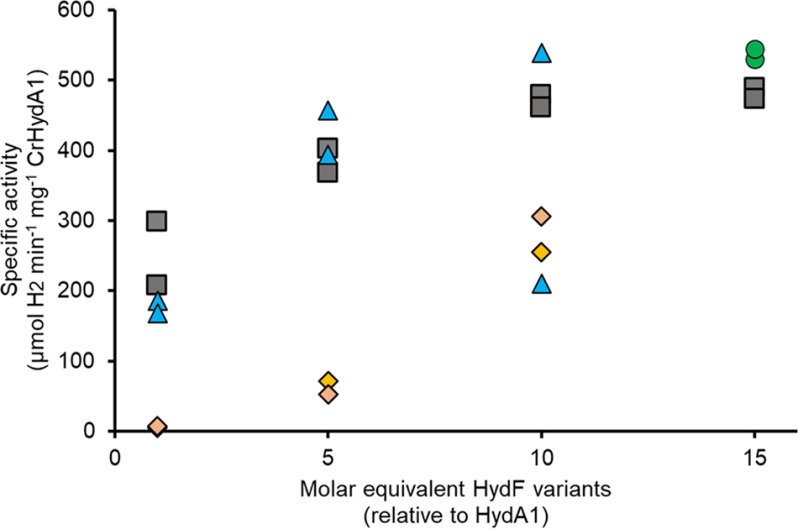
**Influence of the different domains of HydF on the transfer of the precatalyst to apo-HydA1.** A representative titration experiment in which [4Fe-4S]-HydA1 (8 nm) was titrated with 8–120 nm of holo-HydF (*gray squares*), 8–80 nm holo-HydFΔD (*blue triangles*), and 8–80 nm holo-HydFΔDG (*orange diamonds*). The extent of HydA1 activation was determined by calculating the resulting specific activity. 15-Fold molar excess (120 nm) of [2Fe]^adt^ complex was used as a positive control (*green circles*). Individual data points are indicated, see also Fig. S7. The maturation reactions were performed in 100 mm K-phosphate buffer (pH 6.8), and H_2_ evolution was initiated via addition of dithionite and MV^2+^ after 15 min.

The capacity of the different forms of [2Fe]^adt^-loaded HydF, *i.e.* the full-length holo-HydF, holo-HydFΔD, and holo-HydFΔDG, to release the precatalyst and activate HydA1 was evaluated using hydrogen evolution assays. Solutions of [4Fe-4S]-HydA1 were titrated with increasing amounts of the different forms of holo-HydF, and the extent of HydA1 activation was determined by monitoring the activity of the hydrogenase enzyme in the presence of Na-dithionite and methyl viologen (14, 18, 19). Complete activation was achieved after addition of 10 equivalents of holo-HydF relative to CrHydA1, in agreement with earlier reports (Fig. 7, *gray squares*) (14). In the case of holo-HydFΔD, a very similar trend was observed, with high activities observed already after 5 equivalents and complete activation upon addition of 10 equivalents of holo-HydFΔD (Fig. 7, *blue triangles*, and Fig. S7). Holo-HydFΔDG also generated an active HydA1 enzyme but the efficiency was lower, with an activity that reached 50–60% after the addition of 10 equivalents of holo-HydFΔDG (Fig. 7, *orange diamonds*). Thus, whereas removing the dimerization domain appeared to have a negligible effect on the reaction, deleting both the GTPase domain and the dimerization domain had a negative effect on the capacity of HydF to activate the hydrogenase despite the observed similarities in the binding of the [2Fe]^adt^ precatalyst. Whether all three forms of HydF activate HydA1 through the same mechanism remains to be firmly established. Still, it is clear that both truncated forms of HydF retain the capacity to bind the precatalyst in a reversible function.

## Discussion

Here, we have reported on the complex formation between HydF and HydA and highlighted the importance of cofactor content on the interaction. It was recently reported that the isolated dimeric form of HydF is more efficient at activating HydA than the isolated tetrameric form, but there was no direct evidence on the quaternary structure of the HydF–HydA protein complex (32). The data presented here support the conclusion that HydF also retains its dimeric form in the complex with HydA, whereas no interaction complex between HydA and tetrameric HydF was discernable. In contrast to HydF interactions with HydG and HydE, the binding of HydF to HydA is very weak, explaining the lack of any apparent GTP dependence during the transfer of the precatalyst from HydF to HydA (13, 17). Moreover, as shown in Fig. 4, an important factor for the interaction appears to be the presence of metal cofactors on HydF. Considering the anionic nature of both the [4Fe-4S] cluster as well as the [2Fe]^adt^ complex, these cofactors are expected to generate a surface-exposed negatively charged patch, which might help in directing the precatalyst toward the positively charged tunnel leading into the active site in the nonmature form of HydA (3, 38, 39). However, as efficient H-cluster assembly is achievable under maturase-free conditions, both *in vitro* and *in vivo*, the interaction does not appear to be critical in priming HydA for accepting the precatalyst (29, 31, 39–41). Our studies of HydA1 maturation utilizing truncated forms of HydF further underscore this point. In all forms of HydF, the binding of the precatalyst was reversible, as evident from the formation of active hydrogenase regardless of truncation. It cannot be fully ruled out that the [2Fe]^adt^ complex is released from the protein prior to the interaction with HydA. Still, removal of the dimerization domain did not appear to influence the precatalyst transfer reaction compared to that with the full-length HydF protein. Thus, the dimeric nature of HydF is arguably rather relevant upstream in the maturation process, *i.e.* in the interaction with HydG and/or HydE. Moreover, the apo-hydrogenase could clearly be activated even by HydFΔDG, lacking both the GTPase and the dimerization domain, albeit with lower efficiency. More importantly, the observation that both HydFΔD and HydFΔDG are capable of binding the [2Fe]^adt^ precatalyst underscores that the cofactor is located in the FeS domain of HydF, as previously suggested from spectroscopy and point mutants (14, 18, 20). Moving forward, these simplified forms of HydF can provide suitable scaffolds for the preparation of artificial hydrogenases and a more straightforward route toward obtaining X-ray crystallographic data on the nature of the precatalyst on HydF.

## Experimental procedures

### General

All chemicals were purchased from Sigma or VWR and used as received unless otherwise stated. The protein purity was verified by 12% hand-cast acrylamide gels (Bio-Rad gel cast system), and a PageRuler Plus prestained protein ladder (ThermoFisher Scientific) was used as a mass reference. The protein concentrations were determined by Bradford reagent (ThermoFisher Scientific) using BSA as a protein standard. The presence of iron-sulfur clusters in the reconstituted proteins was monitored by UV-visible spectroscopy, using the extinction coefficient ε_410_= 15,000 m^−1^ cm^−1^ (12), and EPR spectroscopy. The iron content was quantified by a microassay based on a methodology previously reported by Fish (42). In short, commercially available Fe(II) standard solution in acid (Sigma) at 1005 µg/µl concentration was used for the calibration curve. Both the calibration curve points and the proteins were diluted to a final volume of 65 µl. To release the Fe from the proteins, 45 µl 1 m perchloric acid was added to each sample. The acid treatment was carried out at room temperature for 15 min, followed by 15 min of centrifugation at 4 °C and 14,000 × *g*. 90 µl from the resulting supernatant was transferred to a clean microcentrifuge tube, and 72 µl of 1.7-mg/ml disodium bathophenanthroline-disulfonate trihydrate was added. The pH was adjusted with 27 µl ammonium acetate (3× dilution of a saturated stock solution, as described in reference 42) and 36 µl of ascorbate solution (36 mg/ml) was used as a reductant. The additions were followed by vigorous mixing and incubation for 15 min at room temperature. The absorbance spectra of the samples were collected with a Cary UV-visible spectrophotometer. The [2Fe]^adt^ complex was prepared as previously described (43, 44). All anaerobic work was performed in MBRAUN gloveboxes ([O_2_] < 10 ppm). Anaerobic UV-visible spectroscopy was performed in the glovebox using an AvaSpec-ULS2048-USB2-UA-50, Avantes Fiber Optic UV/VIS/NIR spectrometer. Aerobic UV-visible spectroscopy was performed using an Agilent Cary 50 UV-visible spectrometer.

### Construction of truncated HydF proteins

To construct the truncated forms of HydF, the *T. maritima* HydF (TmHydF)-encoding gene was codon optimized and cloned into pET19b vectors by GenScript. This construct was used as the DNA template for the mutagenesis. Details on the sequence and primers are shown in the supporting information.

### HydFΔDG (the FeS cluster domain of HydF fused with an N-terminal MBP tag)

The FeS cluster domain-encoding gene fragment was amplified by PCR using the codon-optimized TmHydF-encoding gene as the template. The PCR product was isolated from an agarose gel and digested with EcoRI and BamHI restriction enzymes. The digested fragment was ligated into an EcoRI-BamHI-digested pMAL-c4x plasmid, in frame with the maltose-binding protein (MBP)-encoding gene. This was followed by transformation into chemically competent DH5α *E. coli* cells. Positive colonies were selected via colony PCR. The positive colonies were used for plasmid purification, and the construct was transformed into Rosetta (II) *E. coli* cells.

### HydFΔD (the FeS cluster domain and the GTPase domain of HydF)

To isolate the dimerization domain-free HydF-encoding gene, the full-length vector was amplified by inverse PCR upstream and downstream of the dimerization domain using the codon-optimized TmHydF-encoding gene as the template. The PCR product was isolated from an agarose gel, and it was phosphorylated by polynucleotide kinase treatment (PNK). The PNK-treated fragment was ligated with T4 ligase and then transformed into chemically competent DH5α *E. coli* cells. Positive colonies were selected via colony PCR. The positive colonies were used for plasmid purification, and the construct was transformed into Rosetta (II) *E. coli* cells.

### Protein expression and purification

The expression and purification of His-tagged HydA from *Chlamydomonas reinhardtii* (HydA1) was performed as previously described (4, 29).

The expression and purification of HydF from *T. maritima* was performed as previously described, with minor modifications (30). Rosetta (II) competent cells were transformed with a *T. maritima* HydF-encoding expression vector (kindly provided by Prof. Marc Fontecave, Collège de France/CEA Grenoble), and the positive clones were selected based on kanamycin resistance. A 100-ml preculture was prepared the day before the preparative-scale culture. The cells were inoculated into premixed LB media (Sigma-Aldrich) complemented with 25 mm phosphate buffer, pH 7.6, and 5% (w/v) glucose. The cell cultures were incubated at 37 °C until the optical density (OD) of the cultures at 600 nm reached 0.4–0.6. The expression of HydF was induced with 0.5 mm IPTG (isopropyl β-d-1-thiogalactopyranoside) overnight at 16 °C. Cells were harvested by centrifugation (4500 rpm, 4 °C, 10 min). The cell pellet was resuspended in a solution containing 150 mm NaCl and 50 mm Tris-HCl, pH 8.0, and centrifuged again (5000 rpm, 20 min, 4 °C) to remove the residual media. The cell paste was frozen in liquid nitrogen and kept at −80°C until further use.

The frozen cell paste was thawed and resuspended in a solution containing 150 mm NaCl and 50 mm Tris-HCl, pH 8.0, supplemented with 0.6 mg/ml lysozyme (lyophilized chicken egg white lysozyme, Sigma-Aldrich), 0.03 mg/ml DNase, and 0.03 mg/ml RNase. The volume of the lysis mixture was adjusted to the optical density and the volume of the culture (5 ml lysis buffer·OD^−1^·liter^−1^ culture). The resuspended cells were frozen in liquid nitrogen and thawed at room temperature 3 times. The semilysed cells were sonicated until the solution became clear. To separate the soluble fraction from the unbroken cells and the membrane fraction, the solution was ultracentrifuged (55,000 rpm, 60 min, 4 °C). The supernatant from the ultracentrifugation step was separated into 30-ml aliquots in falcon tubes, which were heated to 86 °C for 8 min to denature the *E. coli* host proteins. The precipitated proteins were separated by centrifugation (15,000 rpm, 15 min, 4 °C). The supernatant was used for ammonium sulfate precipitation, where crystalline ammonium sulfate was added in small portions to the continuously stirred solution at 4 °C. After the final addition of ammonium sulfate (65% saturation concentration), the cloudy solution was gently agitated at 4 °C for 45 min before centrifugation (15,000 rpm, 4 °C, 15 min). The pellet was resuspended in a solution containing 1 M ammonium sulfate, 150 mm NaCl, and 50 mm Tris-HCl, pH 8.0, with a glass Dounce homogenizer, flash-frozen in liquid nitrogen, and kept at −80°C until further use.

A HiLoad^TM^ 16/10 Phenyl-Sepharose HP 1 (20 ml) (GE Healthcare) column was equilibrated with a solution containing 1 M ammonium sulfate, 150 mm NaCl, and 50 mm Tris-HCl, pH 8.0 (buffer A). The filtrated protein solution was loaded onto the column and the flowthrough was collected. The column was washed with equilibration buffer A until the absorbance reached the baseline. A 0–100% B gradient was applied (buffer B was 150 mm NaCl and 50 mm Tris-HCl, pH 8.0). The protein content in the elution fractions was verified using SDS-PAGE gel, and the HydF-containing fractions were collected and concentrated using 30-kDa Centricon filters (Amicon). The purified protein was flash-frozen in liquid nitrogen and stored at −80 °C.

The expression and purification of HydFΔD was performed analogously to a protocol reported for HydA1, with minor modifications (30). Rosetta (II) competent cells were transformed with a HydFΔD-encoding expression vector, and the positive clones were selected based on ampicillin resistance. A 100-ml preculture was prepared the day before the preparative-scale culture. The cells were inoculated into premixed LB media (Sigma-Aldrich) complemented with 25 mm phosphate buffer, pH 7.6, and 5% (w/v) glucose. The cell cultures were incubated at 37 °C until the optical density of the cultures at 600 nm reached 0.4–0.6. The expression of HydFΔD was induced with 0.5 mm IPTG overnight at 16 °C. Cells were harvested by centrifugation (4500 rpm, 4 °C, 10 min). The cell pellet was resuspended in a solution containing 150 mm NaCl and 50 mm Tris-HCl, pH 8.0, and centrifuged again (5000 rpm, 20 min, 4 °C) to remove the residual media. The cell paste was frozen in liquid nitrogen and kept at −80°C until further use.

The frozen cell paste was thawed and resuspended in a solution containing 150 mm NaCl, 25 mm MgCl_2_, 50 mm Tris-HCl, pH 8.0, supplemented with 0.6 mg/ml lysozyme (lyophilized chicken egg white lysozyme, Sigma-Aldrich), 0.03 mg/ml DNase, and 0.03 mg/ml RNase. The volume of the lysis mixture was adjusted to the optical density and the volume of the culture (5 ml lysis buffer·OD^−1^·liter^−1^ culture). The resuspended cells were frozen in liquid nitrogen and thawed at room temperature 3 times. The semilysed cells were sonicated until the solution became clear. To separate the soluble fraction from the unbroken cells and the membrane fraction, the solution was ultracentrifuged (55,000 rpm, 60 min, 4 °C). The supernatant from the ultracentrifugation step was loaded onto a nickel-nitrilotriacetic acid column equilibrated with buffer containing 75 mm HEPES (pH 7.0), 150 mm NaCl, 25 mm MgCl_2_. The flowthrough was collected and the column was washed extensively until the baseline reached nearly zero. This washing step was followed by a washing step with buffer containing 75 mm HEPES (pH 7.0), 150 mm NaCl, 25 mm MgCl_2_, 50 mm imidazole until the nonspecifically bound proteins were eluted and the UV absorbance reached the baseline again. This step was followed by a linear gradient elution from 50–500 mm imidazole in buffer containing 75 mm HEPES (pH 7.0), 150 mm NaCl, 25 mm MgCl_2_. The HydFΔD-containing elution fractions were collected and concentrated with 30-kDa Amicon Centricon filters. The purified protein was flash-frozen in liquid nitrogen and kept at −80 °C until further use.

The expression and purification of HydFΔDG was performed analogously to the protocol for *Clostridium acetobutylicum* HydF, with minor modifications (19). Rosetta (II) *E. coli* competent cells were transformed with a HydFΔDG-encoding expression vector, and the positive clones were selected based on ampicillin resistance. A 100-ml preculture was prepared the day before the preparative-scale culture. The cells were inoculated into premixed LB media (Sigma-Aldrich) complemented with 25 mm phosphate buffer, pH 7.6, and 5% (w/v) glucose. The cell cultures were incubated at 37 °C until the optical density of the cultures at 600 nm reached 0.4–0.6. The expression of HydFΔDG was induced with 0.5 mm IPTG overnight at 16 °C. Cells were harvested by centrifugation (4500 rpm, 4 °C, 10 min). The cell pellet was resuspended in a solution containing 150 mm NaCl and 50 mm Tris-HCl, pH 8.0, and centrifuged again (5000 rpm, 20 min, 4 °C) to remove the residual media. The cell paste was frozen in liquid nitrogen and kept at −80 °C until further use.

The frozen cell paste was thawed and resuspended in a solution containing 300 mm KCl and 100 mm Tris-HCl, pH 8.0, 25 mm MgCl_2_, 5% glycerol supplemented with 0.6 mg/ml lysozyme (lyophilized chicken egg white lysozyme, Sigma-Aldrich), 0.03 mg/ml DNase, and 0.03 mg/ml RNase. The volume of the lysis mixture was adjusted to the optical density and the volume of the culture (5 ml lysis buffer·OD^−1^·liter^−1^ culture). The resuspended cells were frozen in liquid nitrogen and thawed at room temperature 3 times. The semilysed cells were sonicated until the solution became clear. To separate the soluble fraction from the unbroken cells and the membrane fraction, the solution was ultracentrifuged (55,000 rpm, 60 min, 4 °C). The supernatant from the ultracentrifugation step was loaded onto a StrepTrap column equilibrated with buffer containing 300 mm KCl and 100 mm Tris-HCl, pH 8.0, 25 mm MgCl_2_, 5% glycerol. The flowthrough was collected and the column was washed extensively until the baseline reached nearly zero. This washing step was followed by a second washing step with buffer containing 300 mm KCl and 100 mm Tris-HCl, pH 8.0, 5% glycerol until the nonspecifically bound proteins were eluted and the UV absorbance reached the baseline again. This step was followed by a 10-column-volume elution step with 100 mm Tris-HCl, pH 8.0, 300 mm KCl, 25 mm MgCl_2_, 5% glycerol, 2.5 mm desthiobiotin. The HydFΔDG-containing elution fractions were collected and concentrated with 30-kDa Amicon Centricon filters. The purified protein was flash-frozen in liquid nitrogen and kept at −80 °C until further use.

### Preparation of apo-proteins

Residual metals bound to the as-isolated HydF, HydA1, HydFΔD, and HydFΔDG proteins were removed under strict anaerobic conditions by treating the proteins with a 10-fold molar excess of EDTA at 4 °C in the presence of a 20-fold molar excess of sodium dithionite for 2–4 h.

### Size-exclusion chromatography

The demetallized proteins were centrifuged and loaded onto a previously equilibrated (with a solution containing 150 mm NaCl and 50 mm Tris-HCl, pH 8.0) Superdex-200 size-exclusion chromatographic column and eluted using the same buffer (flowrate, 0.5 ml/min). The elution fractions were collected and concentrated using 30-kDa Centricon filters. The purified proteins were flash-frozen in liquid nitrogen and stored at −80°C.

### In vitro reconstitution and assembly of the holo-proteins

The [4Fe4S] clusters in apo-HydA1, apo-HydF, and the truncated HydF apo-proteins were reconstituted as previously described to generate [4Fe4S]-HydA1, [4Fe4S]-HydF, [4Fe4S]-HydFΔD, and [4Fe4S]-HydFΔDG (29, 30). The reconstituted proteins were aliquoted into PCR tubes and transferred into serum vials before they were flash-frozen in liquid nitrogen outside the glovebox and stored at −80°C until further use.

The incorporation of [2Fe]^adt^ into the reconstituted proteins to generate holo-HydF, holo-HydA1, holo-HydFΔD, and holo-HydFΔDG was performed using a procedure from the literature, with minor modifications. In a standard reaction, [4Fe4S]-HydF (200 μm) in Tris-HCl buffer (150 mm NaCl and 50 mm Tris-HCl, pH 8.0) was incubated with [2Fe]^adt^ (1.44 mm, 12-fold molar excess) under dimmed light conditions at ambient temperature. After 1 h, the reaction was stopped and excess [2Fe]^adt^ removed from the solution via a NAP-25 desalting column equilibrated with 100 mm Tris-HCl, pH 8.0. The purified holo-proteins were aliquoted into PCR tubes and transferred into serum vials before being flash-frozen in liquid nitrogen outside the glovebox and stored at −80°C.

### EPR sample preparation and measurements

EPR samples were prepared under strict anaerobic conditions. The proteins were reduced with a 10-fold molar excess of sodium dithionite, and the reaction was monitored by UV-visible spectroscopy. The samples were transferred into quartz EPR tubes capped with rubber septa and immediately flash-frozen outside the glovebox. The EPR samples were stored in liquid nitrogen until further usage.

The CW EPR measurements were carried out on a Bruker Elexys 500X-band spectrometer using an ER049X SuperX microwave bridge in a Bruker SHQ0601 resonator, equipped with an Oxford Instruments continuous-flow cryostat and an ITC 503 temperature controller (Oxford Instruments). Low temperatures were achieved using liquid helium as the coolant. The spectrometer was controlled by the Xepr software package (Bruker). Standard measuring parameters were 10-G modulation amplitude and 100-kHz modulation frequency. The spectra were averaged over either four or eight scans.

### FTIR spectroscopy

FTIR measurements were performed using a demountable liquid FTIR cell (Pike Technologies) with calcium fluoride windows and a 25-micron Teflon spacer. To prevent leakage and to minimize the sample volume, silicon grease was applied to the spacer before cell assembly.

FTIR spectra were collected with a Bruker IFS 66v/S FT-IR spectrophotometer equipped with a Bruker MCT (mercury–cadmium–telluride) detector. The interferograms were accumulated in the double-sided, forward–backward mode with 750 scans. All measurements were performed at a resolution of 2 cm^−1^. Baseline treatment was carried out using the rubber band method in Opus software.

### GEMMA sample preparation and measurements

To avoid disulfide bridge formation between the apo-proteins, DTT (1 mm final concentration) was added to all samples under strict anaerobic conditions in a glovebox (MBRAUN). The nonvolatile salts were replaced by buffer exchange into 100 mm ammonium acetate (pH 7.6), which was carried out with 30-kDa Centricon filters under strictly anaerobic conditions. The protein solutions were analyzed by UV-visible, EPR, and FTIR spectroscopy before and after buffer exchange to verify protein and cofactor stability (see Fig. S1). After the buffer exchange, samples were aliquoted into PCR tubes before being flash-frozen and stored as described for the reconstituted proteins.

Immediately prior to the measurements, the protein stock solutions were diluted into the 0.02–0.06-µg/µl range in a solution containing 20 mm ammonium acetate and 0.005% (v/v) Tween-20. Each sample was scanned 5 times, and a density of 0.58 g/cm^3^ was used for the conversion from diameter to molecular mass. Samples were loaded with a pressure of 1.7–2 PSI. The absolute peak heights may vary between experiments of the same kind depending on the capillary and capillary pressure used, whereas there is much less variation in the relative peak heights.

The GEMMA system contained the following components: a 3480 electrospray aerosol generator, a 3080 electrostatic classifier, a 3085 differential mobility analyzer, and a 3025A ultrafine condensation particle counter (TSI Corp., Shoreview, MN, USA).

### Protein–protein docking

The crystal structures of *C. reinhardtii* HydA1 (PDB entry 3LX4) and *T. melanesiensis* HydF (PDB entry 5KH0) were obtained from the PDB database (45) and prepared for protein–protein docking using YASARA (46), structure version 18.3.23. HydF was initially prepared by deleting one of the dimers. All missing hydrogens were then added to both structures. An energy minimization was performed inside a water-filled simulation cell (cuboid shape, 10 Å around all atoms) with periodic boundaries using the AMBER14 force field (36). The energy-minimized structures were finally subjected to rigid-body protein-protein docking using ClusPro 2.0 (33–35).

## Data availability

All data discussed are contained within the manuscript or the accompanying supporting information document, except for the influence of time on GEMMA spectra of apo-TmHydF. The latter can be shared on request by the corresponding author.

## Supplementary Material

Supporting Information

## References

[1] Lubitz W., Ogata H., Rüdiger O., and Reijerse E. (2014) Hydrogenases. Chem. Rev. 114, 4081–4148 10.1021/cr4005814 24655035

[2] Esmieu C., Raleiras P., and Berggren G. (2018) From protein engineering to artificial enzymes - biological and biomimetic approaches towards sustainable hydrogen production. Sustainable Energy Fuels 2, 724–750 10.1039/c7se00582b 31497651 PMC6695573

[3] Mulder D. W., Boyd E. S., Sarma R., Lange R. K., Endrizzi J. A., Broderick J. B., and Peters J. W. (2010) Stepwise [FeFe]-hydrogenase H-cluster assembly revealed in the structure of HydA^ΔEFG^. Nature 465, 248–251 10.1038/nature08993 20418861

[4] Mulder D. W., Ortillo D. O., Gardenghi D. J., Naumov A. V., Ruebush S. S., Szilagyi R. K., Huynh B., Broderick J. B., and Peters J. W. (2009) Activation of HydA1ΔEFG requires a preformed [4Fe-4S] Cluster. Biochem. 48, 6240–6248 10.1021/bi9000563 19435321

[5] Bai Y., Chen T., Happe T., Lu Y., and Sawyer A. (2018) Iron–sulphur cluster biogenesis via the SUF pathway. Metallomics 10, 1038–1052 10.1039/c8mt00150b 30019043

[6] King P. W., Posewitz M. C., Ghirardi M. L., and Seibert M. (2006) Functional studies of [FeFe] hydrogenase maturation in an Escherichia coli biosynthetic system. J. Bacteriol. 188, 2163–2172 10.1128/JB.188.6.2163-2172.2006 16513746 PMC1428129

[7] Rubach J. K., Brazzolotto X., Gaillard J., and Fontecave M. (2005) Biochemical characterization of the HydE and HydG iron-only hydrogenase maturation enzymes from Thermatoga maritima. FEBS Lett. 579, 5055–5060 10.1016/j.febslet.2005.07.092 16137685

[8] Suess D. L. M., Pham C. C., Bürstel I., Swartz J. R., Cramer S. P., and Britt R. D. (2016) The radical SAM enzyme HydG requires cysteine and a dangler iron for generating an organometallic precursor to the [FeFe]-hydrogenase H-cluster. J. Am. Chem. Soc. 138, 1146–1149 10.1021/jacs.5b12512 26764535 PMC4772725

[9] Suess D. L. M., Kuchenreuther J. M., De La Paz L., Swartz J. R., and Britt R. D. (2016) Biosynthesis of the [FeFe] hydrogenase H cluster: a central role for the radical SAM enzyme HydG. Inorg. Chem. 55, 478–487 10.1021/acs.inorgchem.5b02274 26703931 PMC4780679

[10] Kuchenreuther J. M., Myers W. K., Stich T. A., George S. J., NejatyJahromy Y., Swartz J. R., and Britt R. D. (2013) A radical intermediate in tyrosine scission to the CO and CN^−^ ligands of FeFe hydrogenase. Science 342, 472–475 10.1126/science.1241859 24159045

[11] Rohac R., Amara P., Benjdia A., Martin L., Ruffié P., Favier A., Berteau O., Mouesca J.-M., Fontecilla-Camps J. C., and Nicolet Y. (2016) Carbon–sulfur bond-forming reaction catalysed by the radical SAM enzyme HydE. Nat. Chem. 8, 491–500 10.1038/nchem.2490 27102684

[12] Betz J. N., Boswell N. W., Fugate C. J., Holliday G. L., Akiva E., Scott A. G., Babbitt P. C., Peters J. W., Shepard E. M., and Broderick J. B. (2015) [FeFe]-hydrogenase maturation: insights into the role HydE plays in dithiomethylamine biosynthesis. Biochemistry 54, 1807–1818 10.1021/bi501205e 25654171 PMC4839199

[13] McGlynn S. E., Shepard E. M., Winslow M. A., Naumov A. V., Duschene K. S., Posewitz M. C., Broderick W. E., Broderick J. B., and Peters J. W. (2008) HydF as a scaffold protein in [FeFe] hydrogenase H-cluster biosynthesis. FEBS Lett. 582, 2183–2187 10.1016/j.febslet.2008.04.063 18501709

[14] Berggren G., Adamska A., Lambertz C., Simmons T. R., Esselborn J., Atta M., Gambarelli S., Mouesca J. M., Reijerse E., Lubitz W., Happe T., Artero V., and Fontecave M. (2013) Biomimetic assembly and activation of [FeFe]-hydrogenases. Nature 499, 66–69 10.1038/nature12239 23803769 PMC3793303

[15] Shepard E. M., Mus F., Betz J. N., Byer A. S., Duffus B. R., Peters J. W., and Broderick J. B. (2014) [FeFe]-hydrogenase maturation. Biochemistry 53, 4090–4104 10.1021/bi500210x 24878200

[16] Czech I., Silakov A., Lubitz W., and Happe T. (2010) The [FeFe]-hydrogenase maturase HydF from Clostridium acetobutylicum contains a CO and CN-ligated iron cofactor. FEBS Lett. 584, 638–642 10.1016/j.febslet.2009.12.016 20018187

[17] Shepard E. M., McGlynn S. E., Bueling A. L., Grady-Smith C. S., George S. J., Winslow M. A., Cramer S. P., Peters J. W., and Broderick J. B. (2010) Synthesis of the 2Fe subcluster of the [FeFe]-hydrogenase H cluster on the HydF scaffold. Proc. Natl. Acad. Sci. U S A 107, 10448–10453 10.1073/pnas.1001937107 20498089 PMC2890834

[18] Caserta G., Pecqueur L., Adamska-Venkatesh A., Papini C., Roy S., Artero V., Atta M., Reijerse E., Lubitz W., and Fontecave M. (2017) Structural and functional characterization of the hydrogenase-maturation HydF protein. Nat. Chem. Biol. 13, 779–784 10.1038/nchembio.2385 28553946

[19] Németh B., Esmieu C., Redman H. J., and Berggren G. (2019) Monitoring H-cluster assembly using a semi-synthetic HydF protein. Dalton Trans. 48, 5978–5986 10.1039/c8dt04294b 30632592 PMC6509880

[20] Scott A. G., Szilagyi R. K., Mulder D. W., Ratzloff M. W., Byer A. S., King P. W., Broderick W. E., Shepard E. M., and Broderick J. B. (2018) Compositional and structural insights into the nature of the H-cluster precursor on HydF. Dalton Trans. 47, 9521–9535 10.1039/c8dt01654b 29964288

[21] Brazzolotto X., Rubach J. K., Gaillard J., Gambarelli S., Atta M., and Fontecave M. (2006) The [Fe-Fe]-hydrogenase maturation protein HydF from Thermotoga maritima is a GTPase with an iron-sulfur cluster. J. Biol. Chem. 281, 769–774 10.1074/jbc.M510310200 16278209

[22] Cendron L., Berto P., D'Adamo S., Vallese F., Govoni C., Posewitz M. C., Giacometti G. M., Costantini P., and Zanotti G. (2011) Crystal structure of HydF scaffold protein provides insights into [FeFe]-hydrogenase maturation. J. Biol. Chem. 286, 43944–43950 10.1074/jbc.M111.281956 22057316 PMC3243517

[23] Vallese F., Berto P., Ruzzene M., Cendron L., Sarno S., De Rosa E., Giacometti G. M., and Costantini P. (2012) Biochemical analysis of the interactions between the proteins involved in the [FeFe]-hydrogenase maturation process. J. Biol. Chem. 287, 36544–36555 10.1074/jbc.M112.388900 22932901 PMC3476321

[24] Jonna V. R., Crona M., Rofougaran R., Lundin D., Johansson S., Brännström K., Sjöberg B.-M., and Hofer A. (2015) Diversity in overall activity regulation of ribonucleotide reductase. J. Biol. Chem. 290, 17339–17348 10.1074/jbc.M115.649624 25971975 PMC4498072

[25] Rofougaran R., Crona M., Vodnala M., Sjöberg B.-M., and Hofer A. (2008) Oligomerization status directs overall activity regulation of the Escherichia coli Class IA ribonucleotide reductase. J. Biol. Chem. 283, 35310–35318 10.1074/jbc.M806738200 18835811

[26] Meyer J. (2007) [FeFe] hydrogenases and their evolution: a genomic perspective. Cell. Mol. Life Sci. 64, 1063–1084 10.1007/s00018-007-6477-4 17353991 PMC11136429

[27] Girbal L., von Abendroth G., Winkler M., Benton P. M. C., Meynial-Salles I., Croux C., Peters J. W., Happe T., and Soucaille P. (2005) Homologous and heterologous overexpression in Clostridium acetobutylicum and characterization of purified clostridial and algal Fe-only hydrogenases with high specific activities. Appl. Environ. Microbiol 71, 2777–2781 10.1128/AEM.71.5.2777-2781.2005 15870373 PMC1087525

[28] Artero V., Berggren G., Atta M., Caserta G., Roy S., Pecqueur L., and Fontecave M. (2015) From enzyme maturation to synthetic chemistry: the case of hydrogenases. Acc. Chem. Res. 48, 2380–2387 10.1021/acs.accounts.5b00157 26165393

[29] Mészáros L. S., Németh B., Esmieu C., Ceccaldi P., and Berggren G. (2018) In vivo EPR characterization of semi-synthetic [FeFe] hydrogenases. In vivo EPR characterization of semi-synthetic [FeFe] hydrogenases. Angew. Chem. Int. Ed. 57, 2596–2599 10.1002/anie.201710740 29334424 PMC6282530

[30] Berggren G., Garcia-Serres R., Brazzolotto X., Clemancey M., Gambarelli S., Atta M., Latour J.-M., Hernandez H. L., Subramanian S., Johnson M. K., and Fontecave M. (2014) An EPR/HYSCORE, Mossbauer, and resonance Raman study of the hydrogenase maturation enzyme HydF: a model for N-coordination to 4Fe-4S clusters. J. Biol. Inorg. Chem. 19, 75–84 10.1007/s00775-013-1062-9 24240692 PMC4439245

[31] Esselborn J., Lambertz C., Adamska-Venkates A., Simmons T., Berggren G., Noth J., Siebel J., Hemschemeier A., Artero V., Reijerse E., Fontecave M., Lubitz W., and Happe T. (2013) Spontaneous activation of [FeFe]-hydrogenases by an inorganic [2Fe] active site mimic. Nat. Chem. Biol. 9, 607–609 10.1038/nchembio.1311 23934246 PMC3795299

[32] Shepard E. M., Byer A. S., Aggarwal P., Betz J. N., Scott A. G., Shisler K. A., Usselman R. J., Eaton G. R., Eaton S. S., and Broderick J. B. (2017) Electron spin relaxation and biochemical characterization of the hydrogenase maturase HydF: insights into [2Fe-2S] and [4Fe-4S] cluster communication and hydrogenase activation. Biochemistry 56, 3234–3247 10.1021/acs.biochem.7b00169 28525271 PMC5490485

[33] Kozakov D., Beglov D., Bohnuud T., Mottarella S. E., Xia B., Hall D. R., and Vajda S. (2013) How good is automated protein docking? Proteins 81, 2159–2166 10.1002/prot.24403 23996272 PMC3934018

[34] Kozakov D., Hall D. R., Xia B., Porter K. A., Padhorny D., Yueh C., Beglov D., and Vajda S. (2017) The ClusPro web server for protein-protein docking. Nat. Protoc. 12, 255–278 10.1038/nprot.2016.169 28079879 PMC5540229

[35] Vajda S., Yueh C., Beglov D., Bohnuud T., Mottarella S. E., Xia B., Hall D. R., and Kozakov D. (2017) New additions to the ClusPro server motivated by CAPRI. Proteins 85, 435–444 10.1002/prot.25219 27936493 PMC5313348

[36] Case D. A., Babin V., Berryman J. T., Betz R. M., Cai Q., Cerutti D. S., Cheatham T. E., Darden T. A., Duke R. E., Gohlke H., Goetz A. W., Gusarov S., Homeyer N., Janowski P., Kaus J., et al (2014) Amber 14, University of California, San Francisco

[37] Bortolus M., Costantini P., Doni D., and Carbonera D. (2018) Overview of the maturation machinery of the H-cluster of [FeFe]-hydrogenases with a focus on HydF. Int. J. Mol. Sci. 19, 3118 10.3390/ijms19103118 PMC6212873 30314343

[38] Mulder D. W., Shepard E. M., Meuser J. E., Joshi N., King P. W., Posewitz M. C., Broderick J. B., and Peters J. W. (2011) Insights into [FeFe]-hydrogenase structure, mechanism, and maturation. Structure 19, 1038–1052 10.1016/j.str.2011.06.008 21827941

[39] Lampret O., Esselborn J., Haas R., Rutz A., Booth R. L., Kertess L., Wittkamp F., Megarity C. F., Armstrong F. A., Winkler M., and Happe T. (2019) The final steps of [FeFe]-hydrogenase maturation. Proc. Natl. Acad. Sci. U S A 116, 15802–15810 10.1073/pnas.1908121116 31337676 PMC6689974

[40] Megarity C. F., Esselborn J., Hexter S. V., Wittkamp F., Apfel U.-P., Happe T., and Armstrong F. A. (2016) Electrochemical investigations of the mechanism of assembly of the active-site H-cluster of [FeFe]-Hydrogenases. J. Am. Chem. Soc. 138, 15227–15233 10.1021/jacs.6b09366 27776209

[41] Khanna N., Esmieu C., Meszaros L. S., Lindblad P., and Berggren G. (2017) In vivo activation of an [FeFe] hydrogenase using synthetic cofactors. Energy Environ. Sci. 10, 1563–1567 10.1039/C7EE00135E

[42] Fish W. W. (1988) Rapid colorimetric micromethod for the quantitation of complexed iron in biological samples. Methods Enzymol. 158, 357–364 10.1016/0076-6879(88)58067-9 3374387

[43] Li H., and Rauchfuss T. B. (2002) Iron carbonyl sulfides, formaldehyde, and amines condense to give the proposed azadithiolate cofactor of the Fe-only hydrogenases. J. Am. Chem. Soc. 124, 726–727 10.1021/ja016964n 11817928

[44] Zaffaroni R., Rauchfuss T. B., Gray D. L., De Gioia L., and Zampella G. (2012) Terminal vs bridging hydrides of diiron dithiolates: protonation of Fe_2_(dithiolate)(CO)_2_(PMe_3_)_4_. J. Am. Chem. Soc. 134, 19260–19269 10.1021/ja3094394 23095145 PMC3518320

[45] Berman H., Henrick K., and Nakamura H. (2003) Announcing the worldwide Protein Data Bank. Nat. Struct. Biol. 10, 980 10.1038/nsb1203-980 14634627

[46] Krieger E., and Vriend G. (2014) YASARA View–molecular graphics for all devices–from smartphones to workstations. Bioinformatics 30, 2981–2982 10.1093/bioinformatics/btu426 24996895 PMC4184264

