# Supplementary material for: The maturase HydF enables [FeFe] hydrogenase assembly via transient, cofactor-dependent interactions
Source: J Biol Chem. 2020 Jul 3;295(33):11891–901. doi: 10.1074/jbc.RA119.011419 (PMC7450098; doi:10.1074/jbc.RA119.011419)
Supplement: Supporting Information [file supp_295_33_11891__index.html]

The maturase HydF enables [FeFe] hydrogenase assembly via transient, cofactor-dependent interactions — Studying the HydF-HydA interaction — The maturase HydF enables [FeFe] hydrogenase assembly via transient, cofactor-dependent interactions — Studying the HydF–HydA interaction — Supporting Information 

# The maturase HydF enables [FeFe] hydrogenase assembly via transient, cofactor-dependent interactions

## Supporting Information

- Supporting Information (to be published online) - Supporting information containing additional information with regards to cloning and constructs; Spectroscopic characterization of HydA1 and HydF in ammonium acetate buffer; GEMMA data; In silico structural analysis.
