# Supplementary material for: The maturase HydF enables [FeFe] hydrogenase assembly via transient, cofactor-dependent interactions
Source: J Biol Chem. 2020 Jul 3;295(33):11891–901. doi: 10.1074/jbc.RA119.011419 (PMC7450098; doi:10.1074/jbc.RA119.011419)
Supplement: Supporting Information [file supp_RA119.011419_156374_3_supp_554627_qch4w7.pdf]

Supporting information

[FeFe] hydrogenase maturation and the nature of the HydF-HydA interaction

Brigitta Németh[1], Henrik Land[1], Ann Magnuson[1], Anders Hofer[2] and Gustav Berggren[1,*]

[1]Department of Chemistry - Ångström Laboratory, Uppsala University, Uppsala, Sweden
[2]Department of Medical Biochemistry and Biophysics, Umeå University, Umeå, Sweden

Table of Contents

Supporting information 1

[FeFe] hydrogenase maturation and the nature of the HydF-HydA interaction 1

Cloning 2

Sequence of codon optimized *T. maritima* HydF gene used for truncation 2

HydFΔD mutagenesis primers 2

HydFΔDG mutagenesis primers 2

Amino acid sequences of full length HydF from *T. maritima* and the truncated HydF proteins. 3

Full length TmHydF 3

HydFΔD 3

HydFΔDG – maltose binding protein fusion construct (the MBP derived sequence marked in yellow). 3

Spectroscopic characterization of HydA1 and HydF in Tris and ammonium acetate buffer 4

Predicted structure of TmHydF 5

Selected additional GEMMA spectra 7

Rigid-body protein-protein docking 9

Activation of [4Fe4S]-HydA1 with holo-HydF variants 10

References 10

Cloning

Sequence of codon optimized *T. maritima* HydF gene used for truncation

CATATGCGCC TGCCGGACGC GGGTTTCCGT CGTTACATCG TGGTTGCGGG TCGTCGTAAC GTGGGCAAGA GCAGCTTTAT GAACGCGCTG GTTGGTCAGA ACGTGAGCAT TGTTAGCGAG TATGCGGGCA CCACCACCGA TCCGGTGTAC AAAAGCATGG AACTGTATCC GGTTGGTCCG GTTACCCTGG TGGACACCCC GGGCCTGGAT GATGTTGGCG AGCTGGGCCG TCTGCGTGTG GAAAAGGCGC GTCGTGTTTT CTACCGTGCG GATTGCGGCA TCCTGGTGAC CGACAGCGAG CCGACCCCGT ATGAAGACGA TGTGGTTAAC CTGTTCAAGG AGATGGAAAT CCCGTTTGTG GTTGTGGTTA ACAAAATTGA TGTTCTGGGC GAGAAGGCGG AGGAACTGAA AGGCCTGTAC GAGAGCCGTT ATGAAGCGAA GGTTCTGCTG GTGAGCGCGC TGCAAAAGAA AGGTTTTGAC GATATCGGCA AAACCATCAG CGAAATTCTG CCGGGTCTGG TGCCGCGTGG CAGCGACGAG GAAATCCCGT ACCTGGGTGA CCTGATTGAT GGTGGCGACC TGGTGATCCT GGTGGTTCCG ATTGATCTGG GTGCGCCGAA GGGCCGTCTG ATCATGCCGC AGGTTCACGC GATTCGTGAG GCGCTGGACC GTGAAGCGAT CGCGCTGGTT GTGAAAGAGC GTGAACTGCG TTATGTGATG GAGAACATCG GTATGAAGCC GAAACTGGTT ATTACCGATA GCCAAGTGGT TATGAAGGTT GCGAGCGATG TGCCGGAGGA CGTTGAACTG ACCACCTTCA GCATTGTGGA AAGCCGTTAC CGTGGCGACC TGGCGTATTT TGTTGAGAGC GTGCGTAAGA TCGAGGAACT GGAAGACGGT GATACCGTGG TTATTATGGA GGGTTGCACC CACCGTCCGC TGACCGAAGA TATCGGTCGT GTGAAAATTC CGCGTTGGCT GGTTAACCAC ACCGGTGCGC AGCTGAACTT CAAGGTTATC GCGGGCAAAG ATTTTCCGGA CCTGGAGGAA ATTGAGGGCG CGAAGCTGAT CATTCATTGC GGTGGCTGCG TGCTGAACCG TGCGGCGATG ATGCGTCGTG TTCGTATGGC GAAACGTCTG GGTATCCCGA TGACCAACTA CGGCGTGACC ATTAGCTATC TGCACGGTGT TCTGGACCGT GCGATCCGTC CGTTCCGTGA GGAAGTGAAA GTTTAAGGAT CC

HydFΔD mutagenesis primers

FWD: CAGACCCGGCAGAATTTCGCTGATG

REV: CATATGAGCATTGTGGAAAGCCGTTACCGTGGCGACCTG

HydFΔDG mutagenesis primers

FWD: ATTCCCCTCTAGAAATAATTTTGTTTAACTTTAAGAAGGAGATATACATATGAGCATTGTGGAAAGCCGTTACC

REV: GGATCCTTATTTTTCAAATTGAGGATGTGACCAAACTTTCACTTCCTCACGGAACGGACGGAT

Amino acid sequences of full length HydF from *T. maritima* and the truncated HydF proteins.

Full length TmHydF

MRLPDAGFRRYIVVAGRRNVGKSSFMNALVGQNVSIVSEYAGTTTDPVYKSMELYPVGPVTLVDTPGLDDVGELG
RLRVEKARRVFYRADCGILVTDSEPTPYEDDVVNLFKEMEIPFVVVVNKIDVLGEKAEELKGLYESRYEAKVLLVSALQ
KKGFDDIGKTISEILPGVILVVPIDLGAPKGRLIMPQVHAIREALDREAIALVVKERELRYVMENIGMKPKLVITDSQVV
MKVASDVPEDVELTTFSIVESRYRGDLAYFVESVRKIEELEDGDTVVIMEGCTHRPLTEDIGRVKIPRWLVNHTGAQL
NFKVIAGKDFPDLEEIEGAKLIIHCGGCVLNRAAMMRRVRMAKRLGIPMTNYGVTISYLHGVLDRAIRPFREEVKV

HydFΔD

MRLPDAGFRRYIVVAGRRNVGKSSFMNALVGQNVSIVSEYAGTTTDPVYKSMELYPVGPVTLVDTPGLDDVGELG
RLRVEKARRVFYRADCGILVTDSEPTPYEDDVVNLFKEMEIPFVVVVNKIDVLGEKAEELKGLYESRYEAKVLLVSALQ
KKGFDDIGKTISEILPGSIVESRYRGDLAYFVESVRKIEELEDGDTVVIMEGCTHRPLTEDIGRVKIPRWLVNHTGAQL
NFKVIAGKDFPDLEEIEGAKLIIHCGGCVLNRAAMMRRVRMAKRLGIPMTNYGVTISYLHGVLDRAIRPFREEVKV

HydFΔDG – maltose binding protein fusion construct (the MBP derived sequence marked in yellow).

MKIEEGKLVIWINGDKGYNGLAEVGKKFEKDTGIKVTVEHPDKLEEKFPQVAATGDGPDIIFWAHDRFGGYAQSGL
LAEITPDKAFQDKLYPFTWDAVRYNGKLIAYPIAVEALSLIYNKDLLPNPPKTWEEIPALDKELKAKGKSALMFNLQEP
YFTWPLIAADGGYAFKYENGKYDIKDVGVDNAGAKAGLTFLVDLIKNKHMNADTDYSIAEAAFNKGETAMTINGP
WAWSNIDTSKVNYGVTVLPTFKGQPSKPFVGVLSAGINAASPNKELAKEFLENYLLTDEGLEAVNKDKPLGAVALKS
YEEELVKDPRIAATMENAQKGEIMPNIPQMSAFWYAVRTAVINAASGRQTVDEALKDAQTNSSSNNNNNNNNN
NLGIEGRISEFSIVESRYRGDLAYFVESVRKIEELEDGDTVVIMEGCTHRPLTEDIGRVKIPRWLVNHTGAQLNFKVIA
GKDFPDLEEIEGAKLIIHCGGCVLNRAAMMRRVRMAKRLGIPMTNYGVTISYLHGVLDRAIRPFREEVKVGS

Spectroscopic characterization of HydA1 and HydF in Tris and ammonium acetate buffer

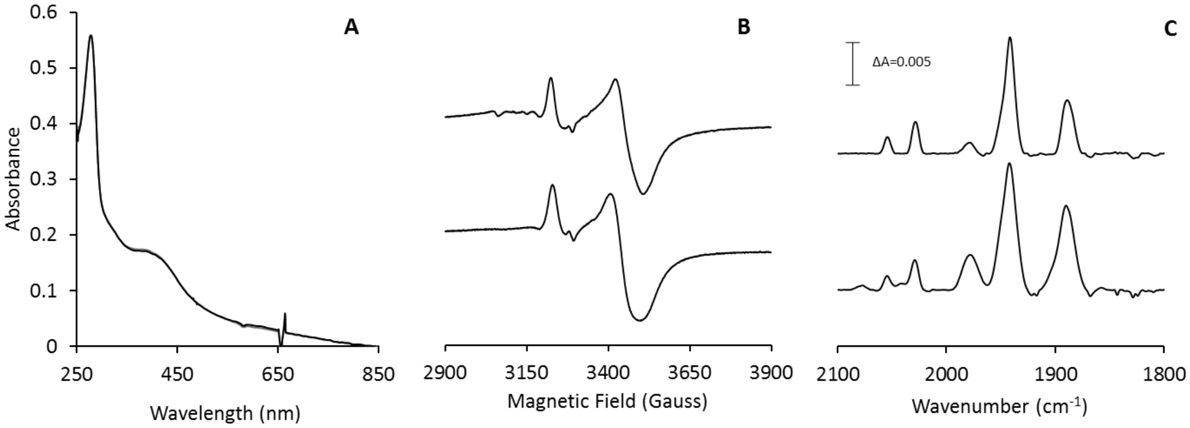

Figure S1. The spectroscopic characterization of HydF proteins in Tris-HCl and ammonium acetate. **A** UV/Vis, **B** Low temperature X-band CW-EPR (bottom Tris-HCl, top ammonium acetate), **C** FTIR (bottom Tris-HCl, top ammonium acetate). EPR spectra are recorded on 200 µM [4Fe4S]-HydF solutions; FTIR spectra are recorded on 2 mM holo-HydF solutions. The buffer composition is either 100 mM ammonium acetate (pH 7), or 50 mM Tris-HCl (pH 8) and 150 mM NaCl

The UV/Visible spectrum of the oxidized [4Fe4S]-TmHydF sample in Tris-HCl pH 8.0 is indistinguishable from the same form of the protein in 100 mM ammonium acetate, pH 7. Similarly, the EPR spectra recorded of the reduced [4Fe4S]-HydF protein show only minor shifts and line broadenings, attributable to a combination of the difference in pH, 7 vs 8, and the change in buffer composition. Still, the relative signal intensity and overall shape of the signal remained constant, further supporting the notion that the FeS cluster was stable also in ammonium acetate buffer. In addition to the stability of the [4Fe4S] in ammonium acetate we also examined the stability of the $[2Fe]^{adt}$ precatalyst. Since the precatalyst is diamagnetic ($Fe_2^{I,I}$) in HydF, the EPR spectra of the chemically reduced holo-HydF proteins is not informative about the stability of the precatalyst. However, the cyanide and carbonyl ligands provide a good spectroscopic probe detectable by FTIR spectroscopy. The FTIR spectrum in the 2100 – 1800 cm^{-1} (carbonyl, cyanide) region of the $[2Fe]^{adt}$ precatalyst coordinating holo-HydF protein in Tris buffer is in agreement with previously published data (1). The holo-HydF protein in ammonium acetate exhibits a practically identical spectrum, confirming that neither the [4Fe4S] nor the [2Fe] subsite is affected by the buffer exchange.

Predicted structure of TmHydF

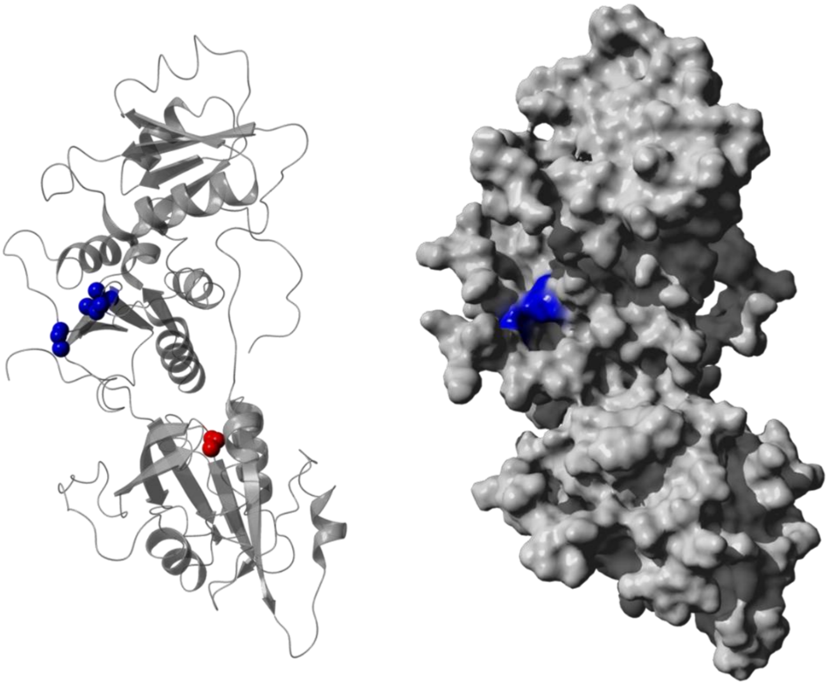

Figure S2. Predicted structure of TmHydF, highlighting the cysteine residues. Cysteines involved in FeS cluster binding marked in blue; cysteine in the GTP:ase domain shown in red. (**Left**) Ribbon structure; (**Right**) surface representation, revealing the buried nature of the GPT:ase domain cysteine. In contrast the to the FeS cluster binding cysteines, the GPT:ase domain cysteine is not visible on the surface.

The model was generated using the RaptorX structure prediction web server.(2) The crystal structure of *T. neapolitana* HydF (PDB ID: 3QQ5) was used as a template. The figure was made in YASARA Structure version 18.3.23.

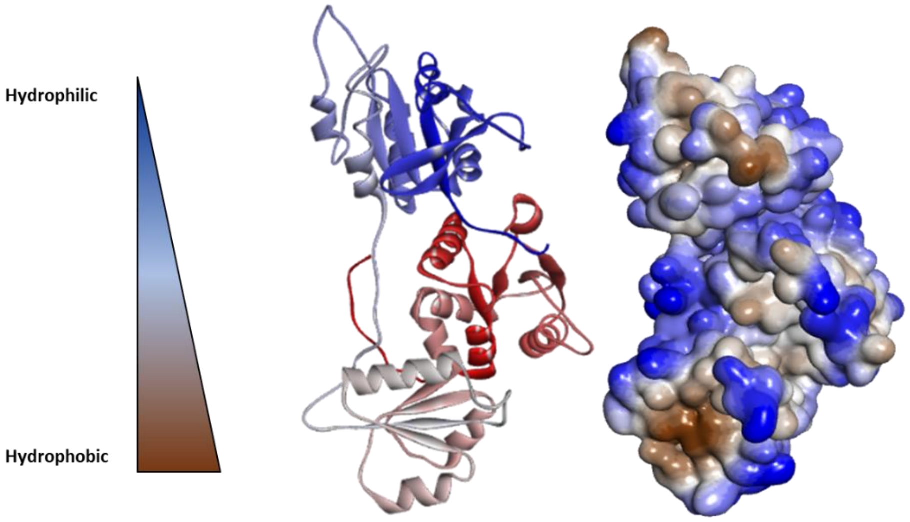

Figure S3. Predicted structure of TmHydF, highlighting the surface properties. The ribbon structure represents the 3 domains of monomeric HydF: Domain 1 – GTP:ase domain (blue), Domain 2 – Dimerization domain, Domain 3 – FeS cluster binding domain (red). In the evaluation software (Discovery Studio, BioDivA) hydrophobic surface was manually applied to show the hydrophobic and hydrophilic surfaces. The model shows that the dimerization domain exhibits a large hydrophobic surface (brown) which is covered by the dimerization domain of another monomer unit in the crystal structure and presumably in solution. The model was generated using the RaptorX structure prediction web server.(2) The crystal structure of *T. neapolitana* HydF (PDB ID: 3QQ5) was used as a template. The figure was made in Discovery studio.

Selected additional GEMMA spectra

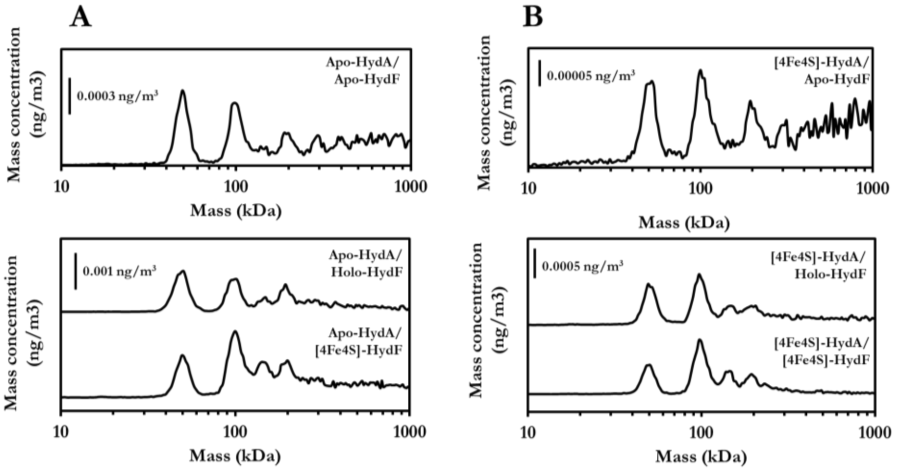

Figure S4. GEMMA spectra recorded on combinations of HydF and HydA1 proteins with different cofactor content. (**A**): apo-HydA1 interaction with different cofactor containing forms of HydF (Top panel: apo-HydF); (bottom panel): holo-HydF; and [4Fe4S]-HydF. (**B**): [4Fe4S]-HydA1 interaction with different cofactor containing forms of HydF (Top panel): apo-HydF; (bottom panel): [4Fe4S]-HydF and holo-HydF. Samples were prepared as described in Figure 4 (main text), except in the case of combinations including apo-HydF. In the latter samples, the ratio of HydA1 to HydF was increased to 2:1 (0.04 mg / mL HydA1 and 0.02 mg / mL HydF). Despite this increase in HydA1 concentration, the 150 kDa interaction peak was still negligible in apo-HydF samples.

Note: The spectra were collected after using different pressure drops, which lead to differences in total signal intensities.

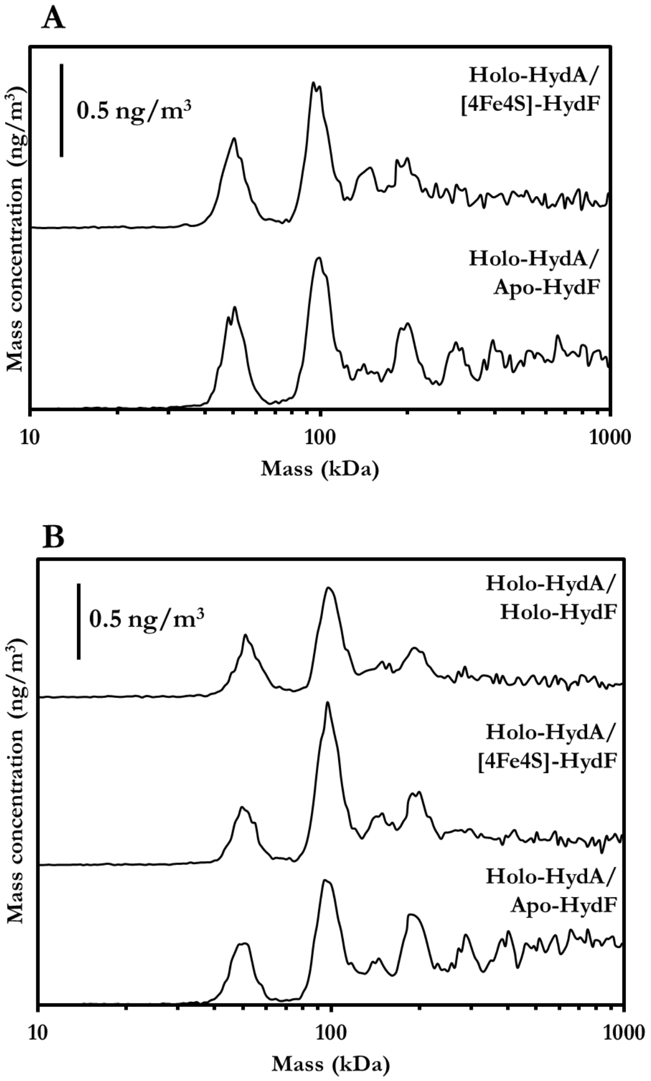

Figure S5. GEMMA spectra recorded on combinations of HydF and HydA1 proteins with different cofactor content. (**A**): holo-HydA1 interaction with different cofactor containing forms of HydF. Top: [4Fe4S]-HydF; bottom: apo-HydF. The protein concentrations were 0.02 mg/ml HydA and 0.02 mg/ml HydF. (**B**): Holo-HydA1 interaction with different cofactor containing forms of HydF. Top: holo-HydF; middle: [4Fe4S]-HydF, bottom: apo-HydF. The protein concentrations were 0.02 mg/ml HydA and 0.04 mg/ml HydF. Samples were prepared as described in Figure 4 (main text). Holo-HydA1 displayed a diminished interaction peak, albeit still clearly discernable in the case of [4Fe4S]-HydF, and also apo-HydF at higher protein concentrations. However, in the latter case the spectra are complicated by the presence of multiple different oligomeric forms of HydF (see main text).

Note: The spectra were collected after using different pressure drops, which lead to differences in total signal intensities.

Rigid-body protein-protein docking

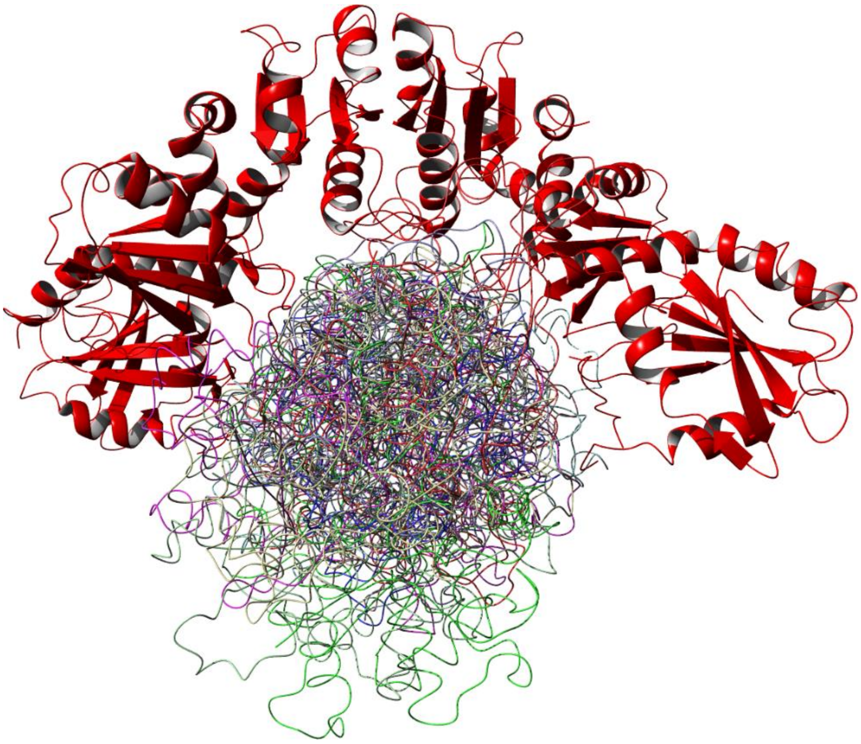

Figure S6. Overlay of the top ten hits from a rigid-body protein-protein docking model of the complex between dimeric [4Fe4S]-HydF (red) and [4Fe4S]-HydA1 (shown in tube format). Models based on reported crystal structures (PDB ID: 5KH0 and 3LX4)(3,4). Docking performed using ClusPro 2.0.

Activation of [4Fe4S]-HydA1 with holo-HydF variants

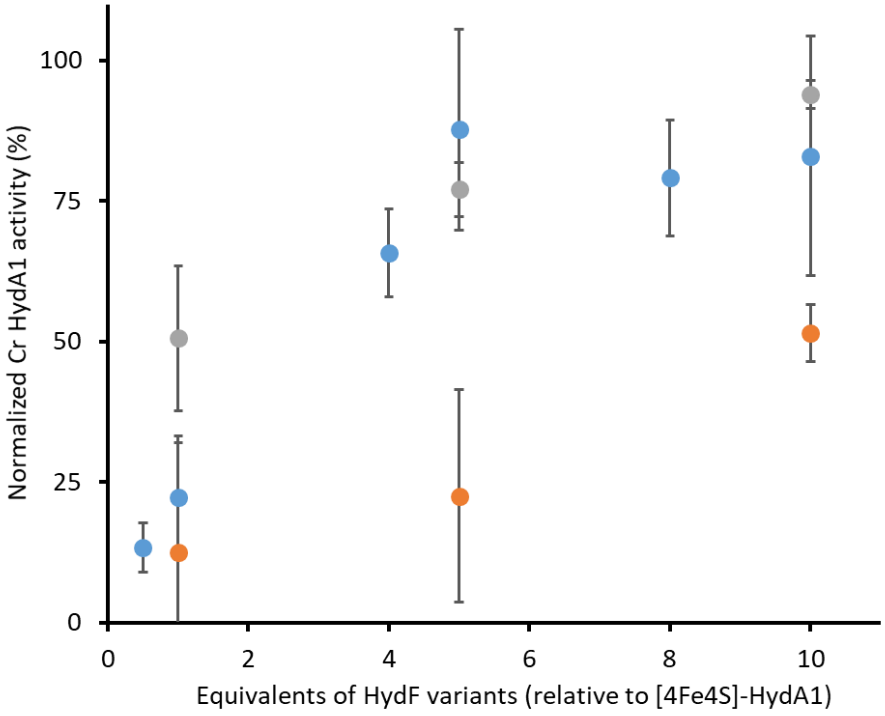

Figure S7. Titration of [4Fe4S]-HydA1 with increasing amounts of holo-HydF variants. Solutions of [4Fe-4S]-HydA1 (8 nM) were titrated with 8-80 nM holo-HydFΔD (blue circles); and 8-80 nM holo-HydFΔDG (orange circles). The extent of HydA1 activation was determined by calculating the resulting specific activity. All data points for the truncated proteins (except 4 and 8 equivalents) represent at least two biological repeats; ± standard deviation is shown as black vertical lines. A titration curve using 8 – 80 nM of holo-HydF is shown for reference (grey circles). *Note*: As the experiments were performed using different preparations of [4Fe4S]-HydA1 the extent of activation is normalized to the maximum activity obtained for that specific preparation (using either holo-HydF or $[2Fe]^{adt}$). The maturation reactions were performed in 100 mM K-phosphate buffer (pH 6.8), and H_2 evolution initiated via addition of dithionite and MV^{2+} after 15 minutes.

References

1. Berggren, G., Adamska, A., Lambertz, C., Simmons, T. R., Esselborn, J., Atta, M., Gambarelli, S., Mouesca, J. M., Reijerse, E., Lubitz, W., Happe, T., Artero, V., and Fontecave, M. (2013) Biomimetic assembly and activation of [FeFe]-hydrogenases. *Nature* **499**, 66-69
2. Källberg, M., Wang, H., Wang, S., Peng, J., Wang, Z., Lu, H., and Xu, J. (2012) Template-based protein structure modeling using the RaptorX web server. *Nat. Protoc.* **7**, 1511-1522
3. Caserta, G., Pecqueur, L., Adamska-Venkatesh, A., Papini, C., Roy, S., Artero, V., Atta, M., Reijerse, E., Lubitz, W., and Fontecave, M. (2017) Structural and functional characterization of the hydrogenase-maturation HydF protein. *Nat Chem Biol* **13**, 779-784
4. Mulder, D. W., Boyd, E. S., Sarma, R., Lange, R. K., Endrizzi, J. A., Broderick, J. B., and Peters, J. W. (2010) Stepwise [FeFe]-hydrogenase H-cluster assembly revealed in the structure of $HydA^{\Delta EFG}$. *Nature* **465**, 248-251
